# KCNK3 Mutation Causes Altered Immune Function in Pulmonary Arterial Hypertension Patients and Mouse Models

**DOI:** 10.3390/ijms22095014

**Published:** 2021-05-09

**Authors:** James D. West, Eric D. Austin, Elise M. Rizzi, Ling Yan, Harikrishna Tanjore, Amber L. Crabtree, Christy S. Moore, Gladson Muthian, Erica J. Carrier, David A. Jacobson, Rizwan Hamid, Peggy L. Kendall, Susan Majka, Anandharajan Rathinasabapathy

**Affiliations:** 1 Division of Allergy, Pulmonary and Critical Care Medicine, Department of Medicine, Vanderbilt University Medical Center, Nashville, TN 37232, USA; j.west@vumc.org (J.D.W.); harikrishna.tanjore@gmail.com (H.T.); amber.l.crabtree@vumc.org (A.L.C.); christy.s.moore@vumc.org (C.S.M.); erica.carrier@vumc.org (E.J.C.); 2 Department of Pediatrics, Vanderbilt University Medical Center, Nashville, TN 37232, USA; eric.austin@vumc.org (E.D.A.); ling.yan@vumc.org (L.Y.); rizwan.hamid@vumc.org (R.H.); 3 Division of Allergy and Immunology, Department of Medicine, Washington University in St. Louis, St. Louis, MO 63110, USA; elise.rizzi@wustl.edu (E.M.R.); peggy.kendall@wustl.edu (P.L.K.); 4 Department of Cancer Biology, Biochemistry and Neuropharmacology, School of Medicine, Meharry Medical College, Nashville, TN 37208, USA; gmuthian@mmc.edu; 5 Department of Molecular Physiology and Biophysics, Vanderbilt University, Nashville, TN 37232, USA; david.a.jacobson@vanderbilt.edu; 6 Division of Pulmonary, Critical Care and Sleep Medicine, Department of Medicine, National Jewish Health, Denver, CO 80206, USA; susanmajka@mac.com

**Keywords:** pulmonary arterial hypertension, inflammation, KCNK3, monocytes, lymphocytes

## Abstract

Loss of function KCNK3 mutation is one of the gene variants driving hereditary pulmonary arterial hypertension (PAH). KCNK3 is expressed in several cell and tissue types on both membrane and endoplasmic reticulum and potentially plays a role in multiple pathological process associated with PAH. However, the role of various stressors driving the susceptibility of KCNK3 mutation to PAH is unknown. Hence, we exposed *kcnk3^fl/fl^* animals to hypoxia, metabolic diet and low dose lipopolysaccharide (LPS) and performed molecular characterization of their tissue. We also used tissue samples from KCNK3 patients (skin fibroblast derived inducible pluripotent stem cells, blood, lungs, peripheral blood mononuclear cells) and performed microarray, immunohistochemistry (IHC) and mass cytometry time of flight (CyTOF) experiments. Although a hypoxic insult did not alter vascular tone in *kcnk3^fl/fl^* mice, RNASeq study of these lungs implied that inflammatory and metabolic factors were altered, and the follow-up diet study demonstrated a dysregulation of bone marrow cells in *kcnk3^fl/fl^* mice. Finally, a low dose LPS study clearly showed that inflammation could be a possible second hit driving PAH in *kcnk3^fl/fl^* mice. Multiplex, IHC and CyTOF immunophenotyping studies on human samples confirmed the mouse data and strongly indicated that cell mediated, and innate immune responses may drive PAH susceptibility in these patients. In conclusion, loss of function KCNK3 mutation alters various physiological processes from vascular tone to metabolic diet through inflammation. Our data suggests that altered circulating immune cells may drive PAH susceptibility in patients with KCNK3 mutation.

## 1. Introduction

KCNK3 is a pH-sensitive potassium channel with expression in many tissues. In 2013, a genetics consortium identified a collection of KCNK3 loss of function mutations as likely causative in a heritable pulmonary arterial hypertension (PAH) family and several idiopathic PAH patients [1]. Since then, several additional patients with KCNK3 mutations have been identified [2,3,4], making this a rare but not singular cause of heritable PAH. As such, PAH has been well established as a lung disease characterized by elevated pulmonary hemodynamics and vascular resistance resulted as a consequence of imbalance between quiescent and hyperproliferative nature of endothelial and vascular smooth muscle cells, which ultimately results in right heart failure and premature death. More specifically, the functional status of these cell types is completely altered in the context of metabolism as seen in BMPR2 (Bone Morphogenetic Protein Receptor Type 2) and KCNK3 mediated PAH [5,6]. In addition, KCNK3 lung protein expression levels are reduced in idiopathic as well as heritable (BMPR2) PAH [7], suggesting that this may be a part of PAH etiology more broadly.

Earlier in vitro experiments on the potassium channel family [8] predicted a role for KCNK3 in modulating pulmonary vascular tone but later investigations performed while this study was in progress demonstrated that in isolated vessels or live mice, there was no obvious impact of KCNK3 on tone or hypoxic response [9,10,11]. Conversely, Kcnk3 KO rats had a slight increase in pressures in response to hypoxia [12], but the effect was subtle—about a 10% increase in RVSP. The Kcnk3 KO rats had a more dramatic response to endothelial injury via monocrotaline, however, and with advanced age developed significantly increased RVSP even without a “second hit” [12] and this preferential increased pulmonary tone in rats explain the absence of KCNK3 mediated contractile property in mice [9,10,11].

KCNK3 is expressed in many tissue types both in the lung and throughout the body, including pulmonary artery smooth muscle cells [13] and endothelium [7], the right ventricle [14] and right and left atria [15], the adrenal gland where it is associated with primary aldosteronism [16], in fat where it regulates thermogenesis [17], and the brain where it is associated with the blood–brain barrier [18]. In T cells, loss of KCNK3 is associated with impaired effector function and protection against autoimmune disorders [19]. In the pancreas, KCNK3 is associated with insulin secretion [20]. In carotid bodies, KCNK3 has been reported to be oxygen and metabolism sensing [21,22], and regulates chemosensory control of breathing [23].

Overall, a variety of potential mechanisms suggested in the literature may predispose KCNK3 mutation to PAH, from tone to metabolism to inflammatory response. However, in the literature to date, it has not been clear how the KCNK3 mutation actually causes PAH and what are the stressors that drive KCNK3 mediated PAH. Hence, the goal of the present study was to determine how KCNK3 mutation drives susceptibility to PAH.

To accomplish this, we examined gene expression in the mesenchymal cells differentiated from inducible pluripotent stem cells derived from patients compared to controls, and subjected total knockout adult KCNK3 mice to different stresses (hypoxic, metabolic and inflammatory) to determine how KCNK3 mutation results in PAH. In addition, we also used mass cytometry tools, identifying newer candidate immune cell types, which would drive future investigation into inflammation mediated PAH in KCNK3 subjects.

## 2. Results

### 2.1. Gene Expression Differences in iPS-Derived Mesenchyme from a PAH Patient with KCNK3 Mutation Compared to Controls

**Figure 1:**
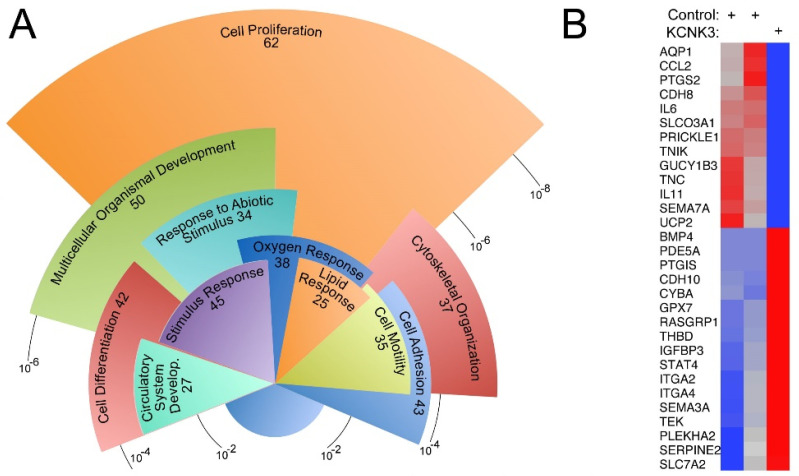
Expression arrays comparing iPSC-derived mesenchyme from a patient carrying a KCNK3 mutation to two controls. (**A**) Overrepresented gene ontology groups from 242 genes with 2-fold changed expression (up or down) in the KCNK3 patient compared to controls. Radius corresponds to multiple-comparisons adjusted *p*-value; angular width corresponds to number of genes. (**B**) Heat map of 30 genes whose pathways had been previously implicated in pulmonary arterial hypertension, related to vascular tone, metabolism, and inflammatory cell recruitment or adhesion. Red indicates higher expression, blue lower expression.

Inducible pluripotent stem cells derived from skin fibroblasts were from two healthy controls and one heritable PAH patient with a KCNK3 mutation. After differentiation into mesenchyme (verified by cell surface markers, Table 1), RNA was collected from each and expression assessed using Affymetrix Human Genome 1.0 ST arrays. A total of 11,134 probe sets had at least one of the samples with expression level above background noise. Comparing KCNK3 to controls results in 302 probe sets with greater than a twofold change, with a false discovery rate of about 10% estimated by swapping group identifiers. These 302 probe sets (Table S1) corresponded to 242 unique entrez IDs with annotation data, of which 152 fell into a statistically significantly overrepresented gene ontology group (Figure 1A).

The largest and most significantly overrepresented gene ontology group was ‘cell proliferation’. However, the nature of the genes included suggest that the actual proliferative rate is not changed—cells that have increased proliferation have changes in, for instance, mitotic spindle, and DNA synthesis genes, which were not present in these cells. Instead, genes directly involved in regulating proliferation such as cell division cycle 6 (CDC6), cell division cycle 20 (CDC20), cyclin dependent kinase inhibitor 2A (CDKN2A), minichromosome maintenance complex component 7 (MCM7), forkhead box protein M1 (FOXM1) and the associated signaling molecules or transcription factors such as insulin like growth factor binding protein 3 (IGFBP3), FAS cell surface death receptor (FAS), Hes related family BHLH transcription factor with TRPW motif 2 (HEY2), short stature homeobox 2 (SHOX2) were changed. In addition, a fair number of genes with historical relevance to pulmonary hypertension (Figure 1B), such as associated with vascular tone, phospho diesterase enzyme 5 (PDE5a), a target of current therapies is increased; guanylate cyclase 1B3 (GUCY1B3), a receptor for nitric oxide is decreased; the inducible form of prostaglandin-endoperoxide synthase 2 (PTGS2) is decreased, among others related to prostaglandin and nitric oxide synthesis and response. Some of the genes relevant to metabolic problems, we have seen in other pulmonary hypertension models are uncoupling protein 2 (UCP2), its loss is associated with PAH [24], downregulated in KCNK3 cells; cytochrome B-245 alpha chain (CYBA), required for some modes of superoxide production [25] is increased; oxidative stress response gene, glutathione peroxidase 7 (GPX7) [26] is increased; of course, there are 25 other lipid response-related genes (Figure 1A). Next, there are groups of genes associated with inflammatory cell recruitment or adhesion to endothelium such as pleckstrin homology domain containing A2 (Plekha2) and integrin subunit alpha 2 and 4 (ITGA2, ITGA4); cytokines like interleukin 6 (IL6) and chemokine C-C motif ligand 6 (CCL2); mitogen-activated protein kinase (MAPK) pathway genes like TEK receptor tyrosine kinase (TEK) and RAS guanyl releasing protein 1 (RASGRP1).

These gene expression differences in iPS-derived mesenchymal cells suggested that KCNK3 mutation may predispose to pulmonary hypertension through mechanisms previously associated with PAH, including vascular tone, metabolism, or inflammation.

### 2.2. Mice with KCNK3 Knocked Out as Adults Lack Increased Susceptibility to Hypoxia

**Figure 2:**
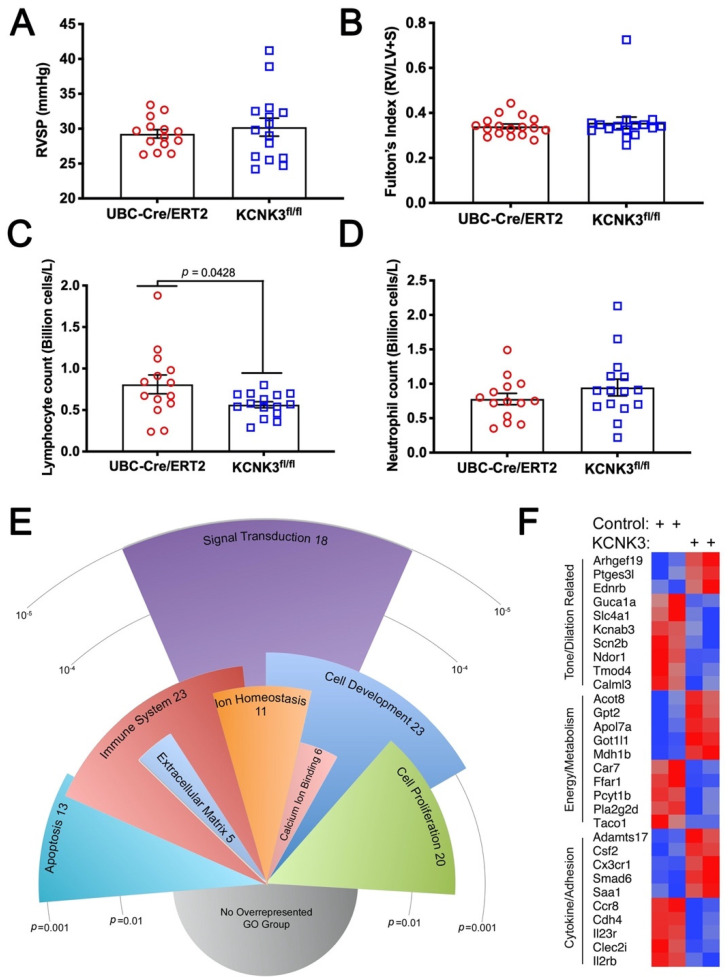
(**A**) Four weeks of normobaric hypoxia (10% O_2_) did not consistently result in higher right ventricular systolic pressures in *kcnk3^fl/fl^* mice. All mice carried the UBC-Cre/ERT gene and were Tamoxifen treated. (**B**) Normobaric hypoxia did not result in right ventricular hypertrophy in *kcnk3^fl/fl^* mice. (**C**) *Kcnk3^fl/fl^* mice had lower lymphocyte count than controls. (**D**) Other elements of the white blood count were normal (neutrophil count shown as an example). (**E**) RNA-Seq comparing lungs from *kcnk3^fl/fl^* mice to controls. Overrepresented gene ontology groups from 138 genes with *p* < 0.05 for change between groups and at least 1.25-fold changed expression (up or down) in the *kcnk3^fl/fl^* mice compared to controls. Radius corresponds to multiple-comparisons adjusted *p*-value; angular width corresponds to number of genes. (**F**) Heat map of 30 genes whose pathways had been previously implicated in pulmonary arterial hypertension, related to vascular tone, metabolism, and inflammatory cell recruitment or adhesion. Red indicates higher expression, blue lower expression.

In order to investigate whether loss of KCNK3 resulted in susceptibility to these ‘second hits’, we bred Ubiquitin-Cre/Ert2 mice, which have universal expression of a tamoxifen-inducible CRE, to mice with loxp sites surrounding the second exon of KCNK3. This system was used because loss of KCNK3 in development could be compensated with KCNK9, with which it frequently dimerizes. We allowed these mice to reach adulthood, and then injected Tamoxifen to both UBC-Cre/ERT2 only mice or UBC-Cre/ERT2 x *kcnk3^fl/fl^* (*kcnk3^fl/fl^*) mice, followed by four weeks of normobaric hypoxia (10% O_2_). Right ventricular systolic pressure (RVSP) in *kcnk3^fl/fl^* mice was indistinguishable from UBC-Cre/ERT2 controls in the hypoxia study (Figure 2A). Unlike the recent report [14], neither in this experiment nor in any of the later experiments did we see right ventricular hypertrophy disproportionate to pressure (Figure 2B). White blood counts in *kcnk3^fl/fl^* mice showed a significant reduction in circulating lymphocytes (Figure 2C), but other elements were unchanged (Figure 2D).

### 2.3. Gene Expression Differences in KCNK3 Mouse Lung by RNA-Seq

To establish whether the gene expression differences seen in our KCNK3 iPSC were also seen in mice, RNA-Seq was performed on sets of pooled RNA (three mice per pool, two male and one female), with two pools each from control and *kcnk3^fl/fl^* mice. In total, 16,606 ensembl gene IDs had a minimum average of 32 reads (log_2_ = 5) in at least one group; this minimum expression cutoff was used to reduce noise. There were 154 unique Entrez IDs with *p* < 0.05 (uncorrected t-test) for difference between control and KCNK3 pools, with expression at least 1.25-fold different (Table S2). In total, 138 of these had associated gene ontology information. A total of 72 of these 138 fell into statistically overrepresented gene ontology groups (Figure 2E).

The source of the RNA was quite different than the Affymetrix arrays described earlier; mouse vs. human; in vivo vs. cell culture; whole lung vs. mesenchyme. Differences in outcome were thus to be expected. Although few of the same genes showed differential regulation, there was substantial overlap in pathways affected (Figure 2F). For instance, while the human iPSC had alteration in PDE5a, GUCY1B3, and PTGS2, the mice had alteration in endothelin receptor type b (Ednrb), guanylate cyclase activator 1A (GUCA1A), and prostaglandin E synthase 3 like (PTGES3l), among others.

### 2.4. Mice with kcnk3 Knocked Out as Adults Lack Susceptibility to Western Diet, but Show Signs of Increased Production and Margination of Lymphocytes

As both the human and mouse gene expression data suggested alterations in energy metabolism, and because we and others have previously shown that Western (high fat) diet increases penetrance of disease in BMPR2 mutants, we stressed *kcnk3* knockout mice with 16 weeks of Western diet.

**Figure 3:**
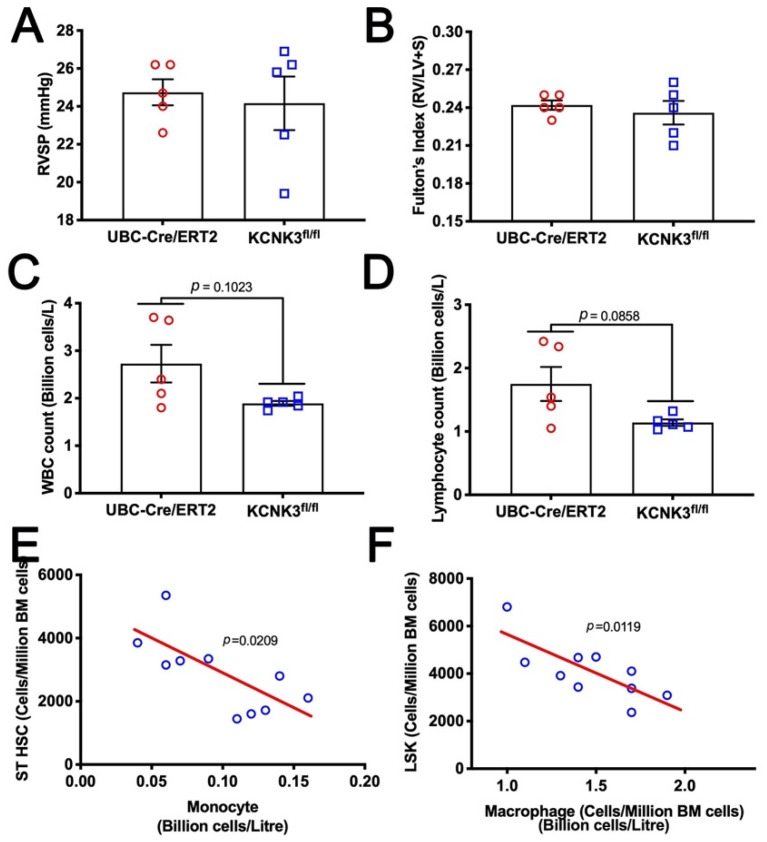
(**A**) High fat diet did not induce pulmonary hypertension in *kcnk3^fl/fl^* mice. All mice carried the UBC-Cre/ERT gene and were Tamoxifen treated. (**B**) High fat diet did not result in right ventricular hypertrophy in *kcnk3^fl/fl^* mice. (**C**) *Kcnk3^fl/fl^* mice trended to lower white blood count than controls. (**D**) *Kcnk3^fl/fl^* mice trended to lower lymphocyte count than controls. (**E**) High short-term hematopoietic stem cells (ST HSC) in the bone marrow correlated to lower levels of monocytes in circulation, implying a high rate of margination and replacement. (**F**) Similarly, high LSK cells in the bone marrow correlate to low macrophage numbers in the circulation. Probability values in panels (**C** and **D**) are by unpaired t-test; (**E** and **F**) are by Pearson correlation probability.

Both male and female adult UBC-Cre/ERT2 and *kcnk3^fl/fl^* mice were treated with tamoxifen, and then given Western diet ad libitum for 16 weeks. *Kcnk3^fl/fl^* and control mice gained 32% and 25% body weight, respectively (not significantly different, although trending in the same direction as prior reports [17]) and were approximately 9 months old at sacrifice. None of the mice—UBC-Cre/ERT2 or *kcnk3^fl/fl^* developed either elevated RVSP (Figure 3A) or right ventricular hypertrophy (Figure 3B). However, as was seen before (Figure 2C), *kcnk3^fl/fl^* mice trended to lower circulating white blood count (Figure 3C) and circulating lymphocyte count (Figure 3D). The lower numbers in this experiment prevent the trend from reaching significance, but the averages are similar to those in the hypoxic experiment. Flow sorting of bone marrow found roughly doubled short term hematopoietic stem cells (ST HSC) in *kcnk3^fl/fl^* compared to control mice (Table 2), coupled with increased Lin^-^Sca1^+^c-Kit^+^ (LSK) cells (multipotent progenitors) and decreased macrophages. Moreover, within the same mouse, a high level of ST HSC cells correlated to a low level of circulating monocytes (Figure 3E) and a high level of LSK cells correlated to a low level of macrophages (Figure 3F). These data strongly suggest that lymphocytes and macrophages are being produced at a high rate within the bone marrow (thus the elevated populations of precursor cells) but are being sequestered into one or more tissues (thus the low level in circulation, and the correlation between the two).

### 2.5. Mice with kcnk3 Knocked Out as Adults Have High Mortality and Develop Pulmonary Hypertension in Response to Low Dose Lipopolysaccharide

As all of our gene expression data, as well as the bone marrow and white blood counts from earlier mouse experiments, suggested potential for susceptibility to an inflammatory ‘second hit’, we tested susceptibility to low dose Lipopolysaccharide (LPS) in *kcnk3* knockout mice.

**Figure 4:**
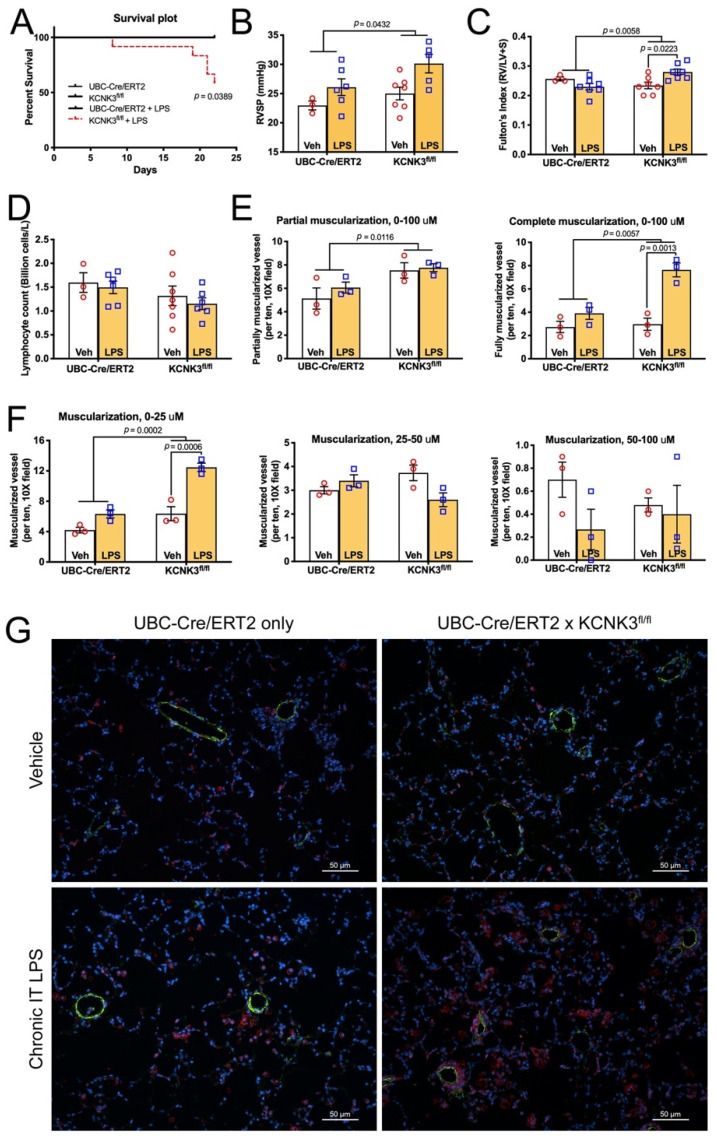
*Kcnk3^fl/fl^* mice have heightened susceptibility to inflammatory insult. (**A**) *Kcnk3^fl/fl^* mice have much higher mortality (5/12 compared to zero) compared to control mice treated with low dose LPS. All mice carried the UBC-Cre/ERT gene and were Tamoxifen treated. (**B**) *Kcnk3^fl/fl^* mice that survived had elevated RVSP. (**C**) *Kcnk3^fl/fl^* mice treated with low dose LPS also had elevated right ventricular hypertrophy, proportional to pressures. (**D**) There were no differences in white blood counts between groups (lymphocytes shown as an example). (**E**) Partially and fully muscularized vessels per field were increased in *kcnk3^fl/fl^* mice. (**F**) Numbers of muscularized vessels were increased in <25 μm but not larger vessels in *kcnk3^fl/fl^* mice. (**G**) Immunofluorescence depicting increased numbers of muscularized vessels (actin, green) and increased CD45 positive inflammatory cells (red) in *kcnk3^fl/fl^* mice.

Adult (average starting age 325 days) UBC-Cre/ERT2 mice (three females, four male) and *kcnk3^fl/fl^* mice (three females, nine male) were treated with tamoxifen, and then given twice weekly injections of low dose LPS for three weeks. During that three weeks, none of the control groups died, compared to five animals in the *kcnk3^fl/fl^* group (Figure 4A). Survivors had significantly elevated RVSP (Figure 4B) and right ventricular hypertrophy (Figure 4C). Unlike previous experiments, white blood counts in this experiment were not significantly different between groups (lymphocytes shown as an example in Figure 4D), but that may have been because of relatively low numbers of survivors and high variability.

*Kcnk3^fl/fl^* mice showed slight but significant increases in partially muscularized vessels and more than tripled numbers of fully muscularized vessels in the lung (Figure 4E). These numbers were driven by increased muscularization of small vessels (<25 μm) with numbers of larger muscularized vessels unchanged (Figure 4F,G).

### 2.6. Kcnk3^fl/fl^ Mice Have Striking Increases in Pulmonary Inflammatory Cells, Multiple Cytokines

**Figure 5:**
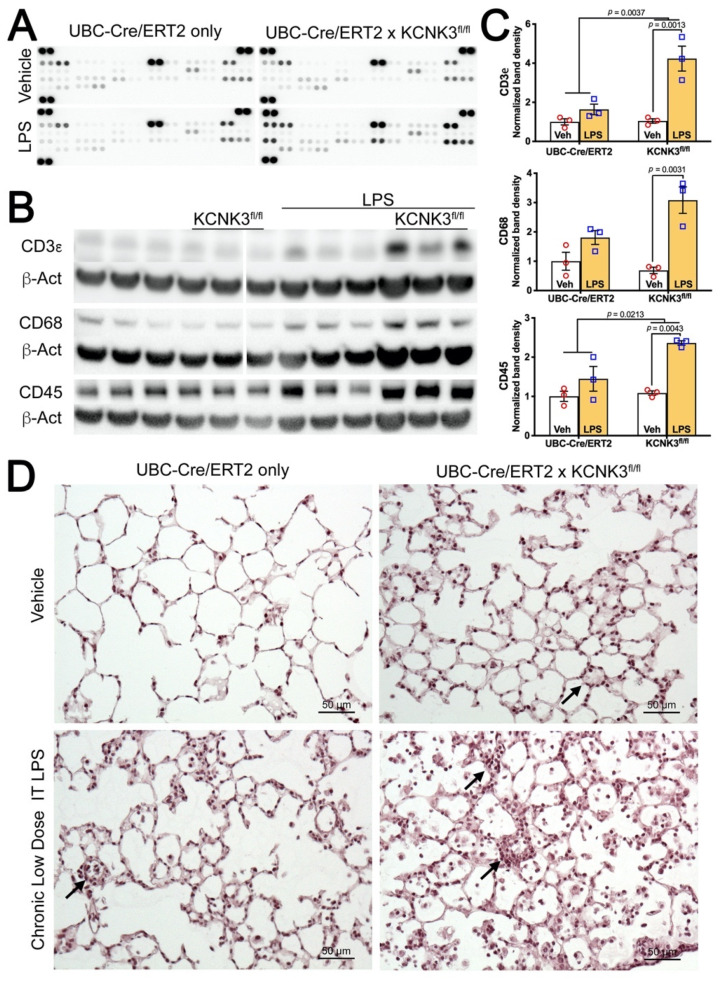
(**A**) Cytokine dot blot arrays indicate stronger response to chronic low dose i.t LPS in UBC-Cre/ERT2 *kcnk3^fl/fl^* homozygous mice than in UBC-Cre/ERT2 only controls. (Quantified in Table 3). Image has had Gamma set to 0.1 to make lower intensity dots more visible. (**B**) Western blot on whole lung shows higher levels of CD3ε^+^ cells (T cells), CD68^+^ cells (macrophages), and CD45^+^ cells (all hematopoietic cells) in UBC-Cre/ERT2 kcnk3^fl/fl^ mice compared to UBC-Cre/ERT2 only controls with LPS. (**C**) Quantification of Western blots; probabilities are by two-way ANOVA with post-hoc pairwise comparisons. (**D**) H&E-stained lungs allowing visualization of increased cellularity in *kcnk3^fl/fl^* mice compared to UBC-Cre/ERT2 only controls with LPS. Black arrows represent the pulmonary vessel.

In addition to increased numbers of muscularized vessels, immunofluorescence makes clear that there is also an increase in numbers of CD45^+^ (pan-circulating) and CD3ε^+^ (T cell) inflammatory cells (Figure 4G and Figure S1). ‘Dot blot’ arrays on whole lung show twofold or greater increases in 14 of 30 cytokines or chemokines, ~10 fold in chemokine C-X-C motif ligand 9 and 10 (CXCL9 and CXCL10) in *kcnk3^fl/fl^* mice compared to controls when treated with LPS (Figure 5A, Table 3). The only cytokine downregulated in *kcnk3^fl/fl^* mice with low dose LPS compared to controls is interleukin 13 (IL-13), which inhibits macrophage activity. No cytokines are increased twofold in controls treated with LPS compared to vehicle treated controls, however, there are several cytokines downregulated (e.g., IL6 down threefold). Many of the cytokines that were downregulated in the controls treated with LPS compared to vehicle treated controls were already downregulated at baseline in the vehicle treated *kcnk3^fl/fl^* mice (IL-6, again). Note that IL-6 was also downregulated in the human iPS cells with KCNK3 mutation (Figure 1B).

As the numbers of inflammatory cells were so high, we attempted to quantify them with Western blots for markers rather than through counts. We found significant increases in the leukocyte marker CD45, the macrophage marker CD68, and particularly the T cell marker CD3ε in *kcnk3^fl/fl^* lungs compared to controls when normalized to β-actin (Figure 5B,C). These inflammatory cells are particularly dense around vessels (Figure 4G, Figure 5D and Figure S1), but are also spread throughout the interstitia and alveoli.

### 2.7. Human PAH Patients with KCNK3 Mutation Show Increased Cytokines and Chronic Immune Cell Activation

**Figure 6:**
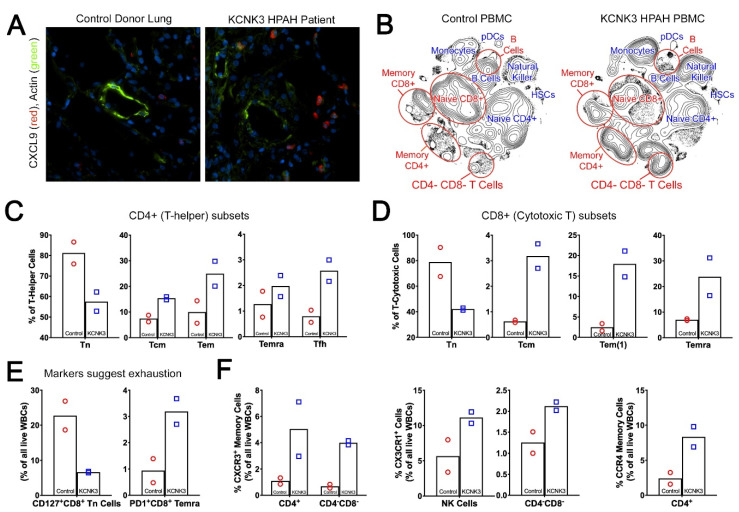
IHC and CyTOF assessment on human tissue samples validate the role of inflammation in driving KCNK3-PAH. (**A**) Immunofluorescence depicting increased numbers CXCL9^+^ cells (red) in the KCNK3 patient lung, around the muscularized vessels (green), 400X magnification. (**B**) 2D density contour expression of mass immunotyping of KCNK3 PBMCs. (**C** and **D**) Naïve T cells are downregulated in KCNK3 PBMCs, compensated through the upregulation of central and memory cells in CD4^+^ and CD8^+^ population. (**E** and **F**) CyTOF findings verify the role of cell mediated immune response in KCNK3 subjects through the dysregulation of CXCR3^+^, CX3CR1^+^, CCR4^+^, CD127^+^CD8^+^ and PD1^+^CD8^+^ cells.

To determine whether the increase in pulmonary inflammation we saw in *kcnk3^fl/fl^* mouse lungs was reflected in human disease, we stained sections from an existing explant lung from a patient with KCNK3 mutation for the cytokine CXCL9, one of the most upregulated cytokines in mice (~10 fold). CXCL9, -10, and -11 induce migration of immune cells to their focal sites, and regulate differentiation of naïve T cells to T helper cells [27]. We found a large increase in CXCL9^+^ cells in the PAH patient with KCNK3 mutation, compared to unused control donor lung (Figure 6A). We also examined plasma of patients with KCNK3 mutation to age and sex matched controls on a multiplex protein assay (Luminex) and found that most cytokines tested were more than twofold upregulated (Table 4). Although the cytokines included on the panel are not a perfect match to those included in the mouse panel, there was broad agreement.

Since myeloid, lymphoid and hematopoietic stem cells prominently drive pulmonary inflammation in *kcnk3^fl/fl^* mice, we did mass immunophenotyping of human peripheral blood mononuclear cells (PBMCs) from patients with KCNK3 mutations compared to age and sex matched controls using a panel of 32 different metal-conjugated antibodies (Table S3) to phenotype marker-specific cell types (Table S4) via mass cytometry (CyTOF). Manually gated concatenated files of cells based on population of interest can be found in Figure S2.

We found striking differences in peripheral recirculating immune cells between healthy controls and KCNK3 HPAH patients (Figure 6B) (demographics in Table S5). These included a relative reduction in naïve CD8^+^ and CD4^+^ T cells as well as naive B cells. In contrast, there were reciprocal increases in memory CD4^+^ and CD8^+^ T cells, as well as double negative CD4^−^/CD8^−^ T cells. The expanded CD4 memory compartment included increased central memory (Tcm), effector memory (Tem), and terminally differentiated effector memory (Temra) cells. (Figure 6C). T follicular helpers (Tfh) were also increased (Figure 6C) but this did not lead to an increase in memory B cells, which were not different (not shown). CD8^+^ subsets showed a similar pattern to CD4^+^ T cells (Figure 6D). The proportions of most innate immune components were also increased in KCNK3 mutant patients, with 2–3 fold, the percentages of classical and nonclassical monocytes, CD16^+^ and CD16^−^ NK cells, and both myeloid and plasmacytoid dendritic cells (Figure S3).

Looking more deeply into markers on these cells, there is evidence of T cell exhaustion. Increase in proportion of cells expressing programmed cell death protein 1 (PD1) and decrease of CD127 (IL-7 receptor α), as found in the KCNK3 HPAH patients, are both associated with T cell exhaustion [28,29] (Figure 6E), suggesting chronic immune activation. Intensity of PD1 staining is also increased, and intensity of CD127 staining in the cells that are still positive is decreased (not shown). The decrease in CD127 has been seen previously in pulmonary hypertension PBMC [30]. Similarly, an increase of PD1 was also previously observed in PH animals [31].

Looking at chemokine C-X-C motif receptor 3 (CXCR3), which is the receptor for CXCL9, the ligand we saw strongly upregulated in whole mouse lung and in the patient lung by IHC in Figure 6A, it is expressed in more of both CD4^+^ and CD4^−^CD8^−^ double negative T cells in KCNK3 HPAH patients (Figure 6F). This has also been seen before in Group 1 PAH [32], and implies an ongoing TH1 response [33].

Similarly, chemokine C-X3-C motif receptor 1 (CX3CR1), the receptor for one of the most significantly increased cytokines seen in Table 4, fractalkine, is expressed in roughly twice as many natural killer (NK) and CD4^−^CD8^−^ cells in KCNK3 HPAH patients as controls (Figure 6F), as well as having higher intensity in the cells it is expressed in (not shown). CX3CR1 is generally implicated in both recruitment as well as self-renewal of T cells [34], and also implies a TH1 polarization of the immune response [35]. An increase in CX3CR1 and its ligand fractalkine has previously been reported in PAH patients [36], although that study primarily found it in CD4^+^ cells, whereas we did not have positivity in CD4^+^ cells in either controls or PAH.

Interestingly, there is also an increase in both number of CD4^+^ T cells with chemokine C-C motif receptor 4 (CCR4) staining (Figure 6F), and intensity of staining in those cells (not shown). These are a non-overlapping population of CD4^+^ T cells compared to those that express higher CXCR3 (not shown). Although also present in TH17 and Tregs, CCR4 is usually a marker of cells with a TH2 orientation [37,38]; presence of CCR4^+^CD4^+^ cells has been associated with pulmonary vascular remodeling [39].

Overall, the CyTOF data supports the idea that KCNK3 patients have both immune activation along multiple axes, as well as immune exhaustion suggesting chronic activation. The CyTOF data is in accord both with the Luminex human plasma cytokine data (Table 4), as well as with the mouse *kcnk3^fl/fl^* lung cytokine data.

## 3. Discussion

**Figure 7:**
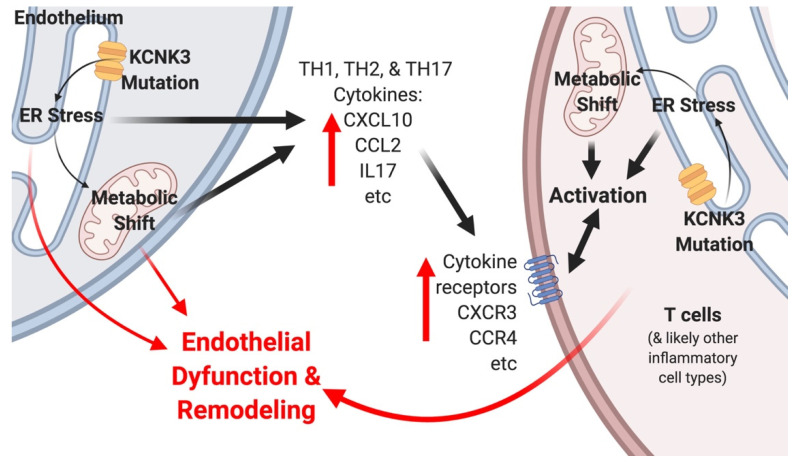
Schematic representation consistent with study outcome, other studies of KCNK3 in pulmonary hypertension, and studies of the role of KCNK3 in the endoplasmic reticulum and other literature. Figure created with BioRender.com (Toronto, Ontario, Canada).

In the present study, gene expression arrays or RNA-seq from human iPS lines with KCNK3 mutation or lungs from mice with KCNK3 knocked out as adults both show a pattern of changes associated with tone, metabolism, and inflammatory cell recruitment (Figure 1 and Figure 2). However, *kcnk3^fl/fl^* mice do not show susceptibility to either hypoxia or Western diet, stresses which bring out an enhanced pulmonary hypertensive phenotype in other models [40,41] (Figure 2 and Figure 3). *kcnk3^fl/fl^* mice do show evidence of increased production and margination of inflammatory cells, however (Figure 2C, Figure 3C–F and Table 2). When they are stressed with a low dose LPS, they have high mortality with the survivors showing elevated RVSP and strongly increased muscularization of small vessels (Figure 4). This is associated with induction of multiple cytokines and chemokines not changed in LPS-treated control mice, and with strong recruitment of inflammatory cells, including macrophages and particularly T cells (Figure 5). Finally, going back to circulating cytokines and mass immunophenotyping of blood cells in PAH patients with KCNK3 mutation, we found that the inflammatory defects were congruent with those found in mice (Figure S3 and Figure 6). In summary, these suggest that KCNK3 mutation results in a predisposition to increased inflammation along multiple axes (Figure 7).

The potential for at least some types of increased inflammation to drive pulmonary hypertension, either directly or as a second hit, has been known for decades. The monocrotaline rat model was described as being a primarily inflammatory model as long ago as 1985 [42], and HHV8 infection was proposed as a trigger for human PAH in 2003 [43]. Since then, multiple cytokines and chemokines have been demonstrated to play a role particularly in muscularization of vessels in PAH. A variety of signaling molecules released by activated endothelium, fibroblasts, monocytes or lymphocytes drive maturation, activation and recruitment of myeloid or lymphoid cells to the site of action [44].

The question arises of how KCNK3 causes the defects, when it does not appear to be regulating tone in a meaningful way. One possibility is that KCNK3 is most important not at the cell membrane, but at the membrane of the endoplasmic reticulum, where it has been reported to regulate endoplasmic reticulum (ER) calcium homeostasis [45] (Figure 7). ER stress has been reported as a feature of pulmonary hypertension for at least 10 years [46], and in fact we saw signs of it in our array analysis of large numbers of PBMC from PAH patients from around that time [47]. This theory, that ER stress in PH is not the result of unfolded protein response, but rather the result of dysregulated calcium, has the advantage that it explains why the hundreds of mutations that even healthy humans have do not result in ER stress, as well as being in accord with BMPR2 suppression or mutation also resulting in potassium channel defects [48]. It also fits well with publications that have shown that ER stress drives metabolic alterations via alterations in mitochondrial function [49,50].

This explanation, which matches both the other literature on KCNK3 mutation in mice and rats [9,10,11,12], as well as the existing PH literature and the data presented in this project, would suppose that KCNK3 mutation primarily impacts ER calcium handling, resulting in ER stress and attendant metabolic alterations. In endothelium, this results in the changes seen in Figure 1 (human iPS) and 2E-F (mouse lung). These include changes in a variety of pathways related to metabolism, proliferation, and apoptosis, which are not themselves sufficient to drive PH, but which also signal to circulating cells. The circulating cells themselves are also impacted by KCNK3 mutation—possibly through the same mechanism. ER stress also impacts immune cell phenotype and immunometabolism [51,52,53].

The nature of the immune reaction is a critical question, however—one needs to know exactly what is happening in order to be able to produce a detailed mechanistic hypothesis that can both be tested and that is amenable to intervention. In earlier studies, Group I pulmonary hypertension was thought to associate with a skew towards TH1/TH17 type inflammation, whereas PH associated with schistosomiasis was associated with TH2 inflammation [54]. In this study, the patient iPS-derived mesenchyme and the mouse lung cytokine arrays from unstimulated mice both expressed lower levels of TH1-oriented cytokines like IL6, which might be similar to the fact, IL6 maintains a stoichiometric balance in shifting the signals from active immune response to suppressive state [55]. One should also note that IL6 signaling mechanism prevents neointimal remodeling in *Schistosoma* associated PH [56], possibly, there might be a dual role for IL6. However, they are apparently very sensitive to insult, because the mice, both in RNA-seq, inflammatory cell recruitment, and cytokines, had an exaggerated response to the low dose LPS, with increases in macrophages, T cells, and a host of TH1, TH2, and TH17 oriented cytokines, also seen in the human luminex arrays and immunophenotyping data. In autoimmune encephalomyelitis, the KCNK3 channel actively mediates T cell proliferation and cytokine production [57,58]; also mediates effector T cell function, similar to our observation seen in Figure 6. Although the strongest signal in the immunophenotyping was in T cells, there were also increases in other types of immune cells. This suggests that the dysregulation actually is not a specific category of immune problem, but rather a problem in common across multiple classes of immune cells. However, because there is a literature on ER stress driving immune cell activation, and on calcium handling problems driving ER stress, it provides points of intervention.

The hypothesis in Figure 7 is consistent with the lack of hypoxia seen in both our KCNK3 model and in several other groups [9,10,11]. If KCNK3′s role in regulating potassium at the membrane is less important than at the ER, then one might not see a change in tone, since the gold particle-transmission electron microscopy demonstrate that KCNK3 channels are trapped in the ER in PH animals [7]. Even the more recent study in rats, which saw a 10% increase in RVSP in response to hypoxia [12], is consistent; this study broadly was consistent with our finding of increased inflammatory response, which might include increased remodeling.

Figure 1 and Figure 2 showed a substantial number of metabolic genes with altered expression in human and mouse with KCNK3 mutation. KCNK3 has published roles in thermogenesis in fat [17], insulin secretion in the pancreas [20], metabolic abnormalities in endothelial and vascular smooth muscle cells [5] and so although our specific results were novel, a metabolic phenotype was within the scope of the literature. Metabolic stress is a clear risk factor for PH in other mouse models [40,41]. In our *kcnk3^fl/fl^* mice, however, even 16 weeks of high fat diet did not result in elevated RVSP. This suggests that although KCNK3 may play a role in regulating elements of energy metabolism, at least in this model, it cannot be pushed all the way to PH just through metabolic stress.

The largest limitation to our work was the limited number of KCNK3 patients we had available; we had tissue available from only one patient in Figure 1, and plasma and PBMC available from only two in Figure 6. However, because KCNK3 is a relatively rare mutation, these problems are unavoidable. Moreover, the limited human data is strengthened by the multiple data types linking mouse and human data. For instance, endothelial CXCL9 and 10 binds to CXCR3 on lymphocytes, NK and dendritic cells executing activation, migration and differentiation of T cells [27]; chemokine C-X3-C motif ligand 1 (CX3CL1), a stimulator of vascular smooth muscle cells from endothelium binds to CX3CR1 on lymphocytes executing chemotactic and leukocyte traffic [59]; macrophage inflammatory protein 1 (MIP1a) and Regulated on Activation, Normal T Cell Expressed and Secreted (RANTES) produced by macrophages and lymphocytes bind to CCR4 on T cells maintaining chemotactic and immune cell homeostasis and obviously, elevated expression of CXCR3 [60] or CX3CR1 [61] or CCR4 [62] are implicated in PAH. A comparative analysis of CyTOF findings between KCNK3 and BMPR2 HPAH patients (data not shown for this ongoing study) suggest that these markers are uniquely associated with KCNK3 mutation, which clearly reinforces our observation and the ensuing conclusion.

The reasons that KCNK3 mutation leads to PAH are important for the patients that have these mutations, of course, but these results hint that the molecular etiology of PAH in these patients may shed light on some features seen in idiopathic patients and patients with other mutations. Understanding in more detail the interplay of metabolism, inflammation, and endothelial dysfunction will be important to PAH of any etiology.

## 4. Materials and Methods

### 4.1. Reagents, Chemicals and Kits

Lipopolysaccharide (LPS) and all other fine chemicals were purchased from Sigma Aldrich (St. Louis, MO, USA). High Fat Diet (HFD) was purchased from Bio-Serv (Flemington, NJ, USA). CD3e (ab49943), CD45 (ab10558), CD68 (ab125212) and beta actin (ab8227) antibodies were purchased from Abcam (Cambridge, MA, USA). Alpha smooth muscle actin (M0851) antibody was obtained from Dako (Santa Clara, CA, USA). Affymetrix Human Genome 1.0 ST arrays were obtained from Thermo Fisher Scientific (Waltham, MA, USA). CXCL9 (PA5-34743) and antibodies (Table S6) used for conventional flow cytometry were obtained from Thermo Fisher Scientific (Waltham, MA, USA). All 32 different metal conjugated antibodies and other reagents (Table S7) used in mass cytometry study were procured from Fluidigm Corporation (San Francisco, CA, USA). Proteome Profiler Mouse Cytokine Array Kit was purchased from R&D systems (Minneapolis, MN, USA). Human High Sensitivity T Cell Panel–Immunology Multiplex Assay kit was purchased from Millipore Sigma (Burlington, MA, USA).

### 4.2. Animal Study Design

All animal protocols in accordance with the guidelines of National Institute of Health were approved by the Institutional Animal Care and Use Committee of the Vanderbilt University Medical Center (M1700106, 1 Aug 2017 and M1700106-01, 22 Jul 2020). All animals were housed in 12:12 h day:night cycle in conventional cages with unlimited access to regular chow and water, unless specified. Tamoxifen-inducible UBC-Cre/ERT2 (ubiquitin) C57BL/6 mice were bred with exon2 floxed KCNK3 mice to generate *kcnk3^fl/fl^*, as explained previously [20]. Briefly, 1 mg of Tamoxifen suspended in olive oil was administered each day for five days through intraperitoneal route to induce recombination. Animals were exposed to high fat diet (HFD, 60% calorie) during these five days. KCNK3 was knocked down to 28 ± 11% (STD) of control levels, as assessed by PCR of genomic DNA, RT-PCR for expression, and RNA-seq expression levels. After 1–3 weeks of Cre induction, animals were used for the following experiments. Both sexes (male and female) were used in all studies. Hemodynamics and all functional outcomes including molecular characterization of *kcnk3^fl/fl^* animals were compared against its controls—UBC-Cre/ERT2.

#### 4.2.1. Hypoxia Study

In this study, all animals, 16–21 weeks old UBC-Cre/ERT2 (*n* = 17) and *kcnk3^fl/fl^* (*n* = 16) mice were exposed to 10% O_2_ normobaric hypoxia for 4 weeks. On termination of the study, RNA was harvested from the lungs and RNASeq study was performed as explained previously [63]. Briefly, RNA from 3 animals were pooled together, 6 animals from each group were pooled as 2 subgroups and subjected to RNA sequencing.

#### 4.2.2. Diet Study

The 19–29 weeks old UBC-Cre/ERT2 (*n* = 5) and *kcnk3^fl/fl^* (*n* = 5) animals were fed on HFD for 4 months. Femurs and tibias were collected from all animals, a single cell bone marrow suspension was made and analyzed for lineage specific hematopoietic markers by flow cytometry as explained previously [64].

#### 4.2.3. LPS Study

The 36–49 weeks old animals were divided into four groups, UBC-Cre/ERT2 + Vehicle (*n* = 3), UBC-Cre/ERT2 + LPS (*n* = 7), *kcnk3^fl/fl^* + Vehicle (*n* = 7) and *kcnk3^fl/fl^* + LPS (*n* = 12). Animals in this study were fed on high energy chow (25.4% fat). In total, 7.5 μg of LPS (Lipopolysaccharide) was intratracheally instilled every 4 days to a cumulative dose 45 μg over 3 weeks. PBS was used as a vehicle control. Animals were provided saline and supplemental gel food and eventually HFD, if they were sick.

### 4.3. Hemodynamic and Right Ventricular Hypertrophy Assessment

After the respective insult (hypoxia, diet or LPS), all animals were subjected to an invasive hemodynamic assessment as explained previously [65]. Subsequently, animals were euthanized, blood was withdrawn, the heart and the lung were exsanguinated, and tissue samples were processed for RNA, protein and histological assessments. Right ventricular hypertrophy or Fulton’s index [RVH = RV/(LV + S)] was calculated as the ratio of wet weight of right ventricle (RV) to the left ventricle + intraventricular septum (LV + S). We also performed a complete blood count analysis.

### 4.4. Western and Dot Blot Analysis

After euthanization, the superior lobe of the right lung was harvested and the protein work, Western and dot blot (cytokine array), was performed as explained previously [65]. CD45, CD68 and CD3ε antibodies were used at 1:500, 1:1000 and 1:1000 dilution. A total of 200 μg of protein was used for dot blot as per manufacturer’s instruction. Details of mouse cytokine array coordinate can be seen in Table S8. All blots were visualized on ChemiDoc MP imaging system (Bio-Rad Laboratories, Hercules, CA, USA) and quantified using Image J software (National Institutes of Health, Bethesda, MD, USA).

### 4.5. Immunohistochemical Analysis (IHC)

After euthanization, the left lobe of the lung was processed, paraffin embedded and IHC staining was performed as explained previously [65]. CD45 (1:100), CD68 (1:100) and CD3ε (1:200) antibodies and hematoxylin-eosin (H&E) staining was used for the assessment of inflammatory cells and alpha smooth muscle actin (1:200) for the assessment of pulmonary vessel muscularization, images were analyzed as explained previously [66]. Similarly, paraffin embedded 5 μm-thick human distal lung sections were stained for CXCL9 (1:200) and imaged.

### 4.6. Human Samples and Study Design

All protocols related to blood or tissue collection from healthy volunteers or patients were approved by the Vanderbilt University Medical Center IRB (Protocol 9401) in accordance with the Declaration of Helsinki. Written consent was obtained from all subjects for tissue or blood collection. Age and sex matched controls were managed for all patient samples. See supplements for more details.

#### 4.6.1. Induced Pluripotent Stem Cells (iPS)

Generation of induced pluripotent stem cells (iPS) and the subsequent cDNA based Affymetrix Human Genome Array was performed as explained previously [67]. Briefly, a fibroblast primary culture cell-line from 3 mm skin biopsy was established, karyotyped and reprogrammed to iPS. Human dermal fibroblast was used as feeder layer for the selection and expansion of iPS cells and those selected were expanded on feeder free mTeSR1 medium. Specificity of iPS cells were assessed on nude mice by in vivo teratoma assay. Subsequently, these cells were differentiated into mesenchymal stem cells (MSC) and utilized for our experimental purpose. MSC lineage was validated by flow cytometry (Table S1). All data acquisition and analysis pertaining human genome were performed as explained previously.

#### 4.6.2. Lungs

Healthy as well as pulmonary hypertension lungs received with approved consent (IRB 9401) were processed for histological studies. The 5 μm thickness distal lung sections were used for the CXCL9 IHC studies.

#### 4.6.3. Blood

In total, 20 mL of blood was collected from each subject—KCNK3-HPAH (*n* = 2) and age/sex matched healthy controls (*n* = 2), through median cubital venous puncture (IRB 9401) in a sodium heparin BD vacutainer tubes (BD Biosciences, San Jose, CA, USA). Immediately, blood was processed through Ficoll-Paque density centrifugation [68], red blood cells lysed through Gibco ACK buffer (Thermo Fisher Scientific, Waltham, MA, USA) and the harvested peripheral blood mononuclear cells (PBMCs) were stored in liquid nitrogen in aliquots of 3–5 × 10^6^ PBMCs/mL, until they were used for CyTOF experiments.

A total of 25 uL of plasma was collected from each KCNK3-HPAH patient and the controls were used for Human High Sensitivity T Cell Panel Luminex Assay (Millipore Sigma, Burlington, MA, USA), as explained previously [63]. Concentration of each analyte was quantified using appropriate standards, as per manufacturer’s instruction.

### 4.7. Mass Immunophenotyping of Human PBMCs (CyTOF)

#### 4.7.1. PBMC Staining Protocol

PBMCs were thawed at 37 °C, resuspended in 10 mL of PBS (without calcium or magnesium) and washed 2× in PBS. PBMCs were then transferred to a 96-well plate and Cisplatin-198 (200 nM, 5 min at room temperature) based viability test was performed. Surface staining of PBMCs was performed by incubating them in surface master mix (Table S7) followed by another incubation in secondary surface master mix (Table S9), fixed in 1.6% PFA and permeabilized for 45 min using Ebioscience FoxP3 fix/perm (Thermo Fisher Scientific, Waltham, MA, USA). Subsequently, cells were incubated in primary and secondary intracellular master mix (Tables S10 and S11) followed by an overnight 4 °C fixation in 1.6% PFA/6.25nM Cell-ID Intercalator. Each antibody staining was carried out 30 min at room temperature with a couple of intermittent washing with 1% BSA in PBS. Antibodies used in the CyTOF study are listed in Supplemental Table S3. Patient demographic details are outlined in Supplemental Table S5.

#### 4.7.2. Data Acquisition and Analysis

Prior to data acquisition, PBMCs were washed twice in PBS, resuspended in MilliQ water (500,000 cells/mL) with 10% by volume EQ Four Element Calibration Beads (Fluidigm) and the resultant suspension was filtered through 35 µm nylon mesh cell-strainer. Immediately, the mass immunophenotype data acquisition was performed in CyTOF Helios (Fluidigm Sciences, Sunnyvale, CA, USA) using CyTOF software version 6.7.1014 for noise reduction and dual count calibration at the Vanderbilt University Medical Center Mass Cytometry Center of Excellence. Resultant FCS files underwent bead-based normalization using the R package Premessa developed by the Parker Institute for Cancer Immunotherapy and were then uploaded to CytoBank (Beckman Coulter) for analysis. Traditional single cell biaxial gating was used to identify live white blood cells, which then underwent a 3000 iteration with perplexity of 100 and theta of 0.5 and the 35 parameter viSNE plots were generated on CytoBank based on Barnes-Hut implementation of the tSNE algorithm. Populations of interest were manually gated based on marker expression (Figure S2).

### 4.8. Statistics

Graph Pad Prism, version 8.4.2 (La Jolla, CA, USA) was used for the statistical analysis. Two-way ANOVA followed by Tukey’s HSD post-hoc analysis was performed to compare the interaction between genotype and treatment. Pearson correlation coefficient was performed to compare the correlation between the variables. Unpaired, parametric t-test with Welch’s correction was used to assess the differences between wild type and KCNK3 animals. Values are represented as means ± SEM, *p* values ≤ 0.05 were considered statistically significant.

## 5. Conclusions

Although KCNK3 potentially plays a role in multiple processes associated with PAH, including proliferation, tone, and metabolism, the mechanism through which it actually leads to susceptibility to PAH is through increased susceptibility to inflammatory second hits. There is strong accord between data from human patients and mutant mice on the nature of the inflammation.

## Figures and Tables

**Table 1:** Cell surface markers of iPS cells by flow cytometry. Inducible pluripotent stem cells (iPS) developed from healthy volunteers and KCNK3-HPAH patients were assessed for their mesenchymal property by flow cytometry. CD73, CD44, CD105 and CD146 are the positive markers demonstrating mesenchymal property.

	Control-1	Control-2	KCNK3
CD73 (%)	100	100	99.9
CD44 (%)	99.9	100	99.8
ABCG2 (%)	1.59	0.032	1.41
CD45 (%)	0.096	0.13	0.19
CD144 (%)	1.00	0.026	0.15
CD105 (%)	99.4	99.9	99.9
CD14 (%)	0.069	0.13	0.03
CD31 (%)	0.00	0.16	0.00
CD34 (%)	1.00	0.00	0.076
CD146 (%)	84.00	99.7	99.5
CD146a (%)	0.014	0.00	0.032

**Table 2:** Assessment of bone-marrow derived myeloid, lymphoid and hematopoietic stem and progenitor cells by flow cytometry. Cell numbers are expressed as total number per million bone marrow cells. LT-HSC, long-term hematopoietic stem cells; ST-HSC, short-term hematopoietic stem cells; MPP, multipotent progenitors; LSK, enrichment of stem cells including LT-HSC, ST-HSC and MPP; MEP, megakaryocyte erythroid progenitor cells; CMP, hematopoietic stem cells^+^CD34^+^FcγR^−^; GMP, hematopoietic stem cells^+^CD34^+^FcγR^+^; B220, CD45R^+^ B cells; Mac^−^1, CD11b^+^ macrophages; Mac^+^Gr1, monocytes.

Cell Type	UBC-Cre/ERT2	*kcnk3^fl/fl^*	*p* Value
LT HSC	228	300	
ST HSC	2007	3727	0.0089
MPP	1039	1701	
LSK	3397	4799	0.0498
MEP%	0.5	0.3	
CMP%	45.1	50.2	
GMP%	47.5	42.4	
B220%	15.9	14.0	
Mac^-^1%	1.6	1.3	0.0308
Mac^+^Gr1%	62.0	60.9	
Gr1%	6.6	6.7	
CD4^+^%	1.4	1.6	
CD8^+^%	1.3	1.3	
CD4^+^CD^+^8%	0.1	0.1	

**Table 3:** Densitometry of cytokine dot blots in whole mouse lung.

	Control		+LPS		Ratio *fl/fl*:Control	
	UBC-Cre/ERT2	*kcnk3^fl/fl^*	UBC-Cre/ERT2	*kcnk3^fl/fl^*	Control	+LPS
C4/C5a	1148	911	826	1145	0.8	1.4
CCL12/MCP-5	157	124	152	590	0.8	3.9
CCL2/MCP-1	1370	1158	489	1917	0.8	3.9
CCL3/MIP-1a	341	217	248	557	0.6	2.3
CCL4/MIP-1b	140	181	120	72	1.3	0.6
CXCL1	781	1370	495	1010	1.8	2.0
CXCL10/CRG-2	1050	526	770	8381	0.5	10.9
CXCL11/I-TAC	496	230	230	398	0.5	1.7
CXCL12/MIP-2	163	165	130	194	1.0	1.5
CXCL13/BCA-1/BLC	489	267	254	1470	0.5	5.8
CXCL9/MIG	389	240	454	4791	0.6	10.6
IL-10	813	312	677	674	0.4	1.0
IL-13	6830	7231	5083	1938	1.1	0.4
IL-16	5305	5922	3640	5415	1.1	1.5
IL-17	463	251	356	1209	0.5	3.4
IL-1ra/IL-1F3	709	701	1101	4138	1.0	3.8
IL-23	557	328	536	1308	0.6	2.4
IL-27	934	488	697	1132	0.5	1.6
IL-3	3269	2358	2533	5727	0.7	2.3
IL-4	3880	3133	3464	5065	0.8	1.5
IL-5	837	95	529	535	0.1	1.0
IL-6	1420	106	374	317	0.1	0.8
IL-7	4281	2131	2610	2610	0.5	1.0
M-CSF	1212	623	506	453	0.5	0.9
RANTES/CCL5	2587	2735	3020	7692	1.1	2.5
SDF-1	2605	2161	1930	4607	0.8	2.4
sICAM-1	8730	7482	8075	7720	0.9	1.0
TIMP-1	4639	3397	3444	13,502	0.7	3.9
TNFa	1932	2628	2699	3094	1.4	1.1
TREM-1	10,167	11,872	6342	8302	1.2	1.3

Numbers are densitometry on dot blots in Figure 5A (arbitrary units). Values that are upregulated or downregulated by more than twofold are highlighted (green and red, respectively).

**Table 4:** Plasma cytokines of PAH patients with KCNK3 mutation. Mean cytokine levels (pg/mL) from the healthy volunteers (*n* = 7) and PAH patients with KCNK3 mutation (*n* = 2). Plasma collected from the subjects were assessed for cytokine levels on multiplex platform. Mean cytokine level of each analyte is the average of duplicates. Data was analyzed by unpaired, parametric t-test with Welch’s correction.

Target	Healthy Volunteers	KCNK3 HPAH Patients	*p* Value
ITAC	21.0 ± 6.6	35.3 ± 4.6	0.31
Fractalkine (CX3CL1)	52.7 ± 4.6	118.3 ± 13.1	<0.01
IFNγ	5.4 ± 0.7	11.8 ± 3.1	0.01
IL10	3.3 ± 1.9	10.0 ± 0.7	0.12
IL13	4.0 ± 1.7	12.3 ± 9.9	0.17
IL17	4.8 ± 0.5	9.5 ± 3.4	0.03
IL1β	0.5 ± 0.0	1.1 ± 0.0	<0.01
IL6	1.0 ± 0.4	2.8 ± 0.5	0.05
MIP1α	2.6 ± 0.8	11.3 ± 9.5	0.08

## Data Availability

All data supporting the results of CyTOF are archived in the following public database: http://flowrepository.org/id/FR-FCM-Z3QC (accessed on 1 January 2019).

## Supplementary Materials

The following supplemental materials are available online: https://www.mdpi.com/article/10.3390/ijms22095014/s1. Figure S1. IHC assessment of CD3ε in mouse lung tissue samples, Figure S2. Live white blood cell viSNE expression heatmaps (concatenated files), Figure S3. CyTOF assessment of human PBMCs suggest the activation of innate immune responses in KCNK3-HPAH patients, Table S1. Gene ontology of iPS microarray, Table S2. RNA-Seq gene expression in whole mouse lung, Table S3. List of Fluidigm and Biolegend antibodies used for mass immunophenotyping, Table S4. Immuno cell type surface markers used in the CyTOF study, Table S5. Demographics of volunteers/patients served in the CyTOF study, Table S6. List of antibodies used in conventional flow cytometry, Table S7. Surface Master Mix, Table S8. Mouse cytokine array coordinates, Table S9. Secondary Surface Master Mix, Table S10. Primary Intracellular Master Mix, Table S11. Secondary Intracellular Master Mix.
