# Supplementary material for: KCNK3 Mutation Causes Altered Immune Function in Pulmonary Arterial Hypertension Patients and Mouse Models
Source: Int J Mol Sci. 2021 May 9;22(9):5014. doi: 10.3390/ijms22095014 (PMC8126011; doi:10.3390/ijms22095014)
Supplement: Supplementary file 1 [file ijms-22-05014-s001.zip › ijms-1189402-SI.pdf]

Online Supplement

KCNK3 Mutation Causes Altered Immune Function in Pulmonary Arterial Hypertension Patients and Mouse Models

James D. West[1], Eric D. Austin[2], Elise M. Rizzi[3], Ling Yan[2], Harikrishna Tanjore[1], Amber L. Crabtree[1], Christy S. Moore[1], Gladson Muthian[4], Erica J. Carrier[1], David A. Jacobson[5], Rizwan Hamid[2], Peggy L. Kendall[3], Susan Majka[6], Anandharajan Rathinasabapathy[1]

[1] Division of Allergy, Pulmonary and Critical Care Medicine, Department of Medicine, Vanderbilt University Medical Center, Nashville, USA
[2] Department of Pediatrics, Vanderbilt University Medical Center, Nashville, USA
[3] Division of Allergy and Immunology, Department of Medicine, Washington University in St. Louis, St. Louis, USA
[4] Department of Cancer Biology, Biochemistry and Neuropharmacology, School of Medicine, Meharry Medical College, Nashville, USA
[5] Department of Molecular Physiology and Biophysics, Vanderbilt University, Nashville, USA
[6] Division of Pulmonary, Critical Care and Sleep Medicine, Department of Medicine, National Jewish Health, Denver, USA

Corresponding author:
Anandharajan Rathinasabapathy PhD
Vanderbilt University Medical Center
1161 21st Ave S
T-1218 MCN
Nashville, TN 37232-2650
phone: 615 343-3311
fax: 615 343-3480
anandharajan.rathinasabapathy@vumc.org

Supplemental Figures

Figure S1. IHC assessment of CD3ε in mouse lung tissue samples

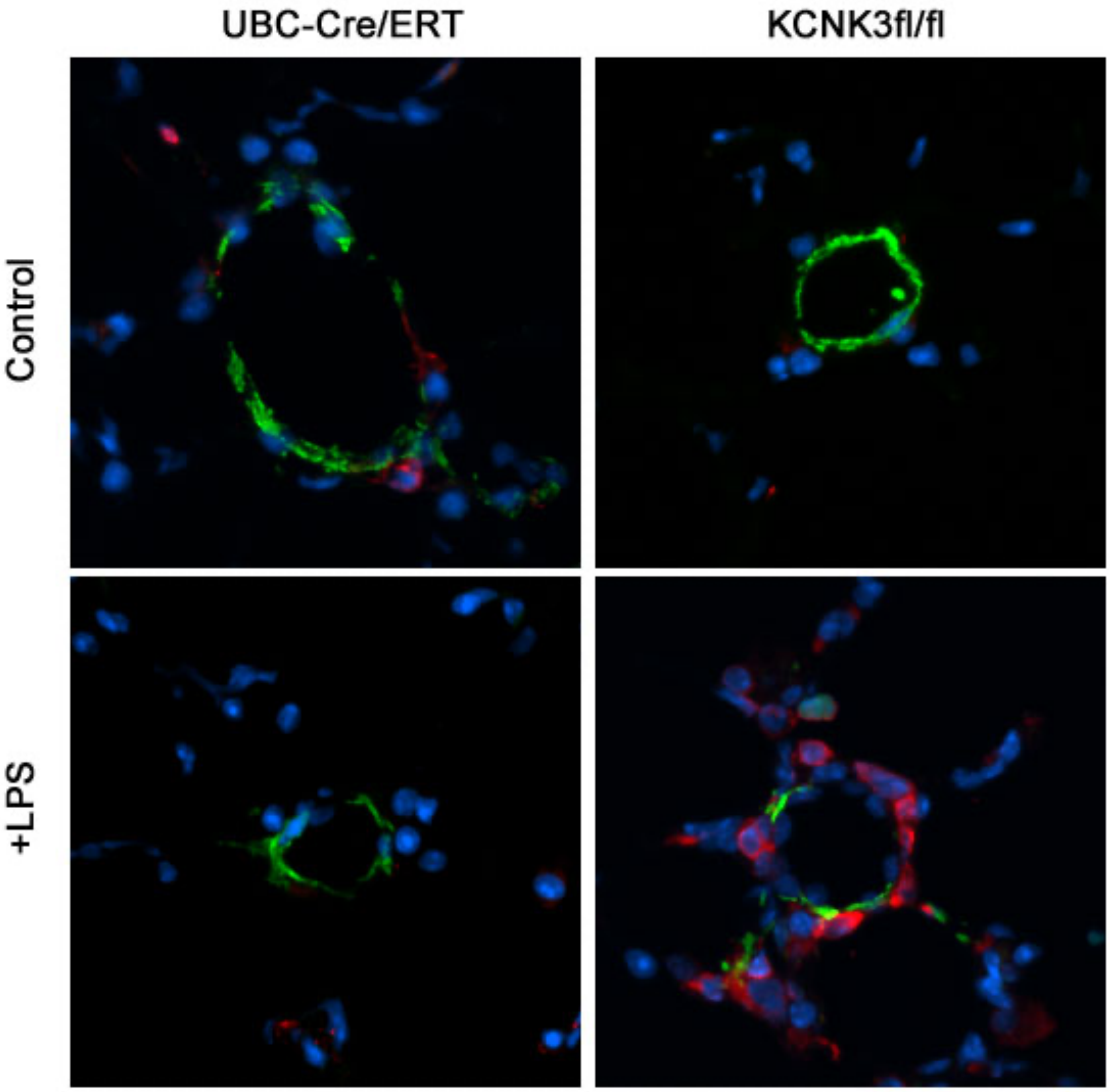

IHC assessment on mouse tissue samples validate the role of inflammation in driving KCNK3-PAH. Immunofluorescence depicting increased numbers of CD3ε^{+} cells (red) around the muscularized vessels (green) in UBC-Cre/ERT2 *kcnk3*$^{fl/fl}$ mice treated with low dose LPS – these are less common in other conditions. Nucleus is stained in blue. Images are magnified at 400X.

Figure S2. Live White Blood Cell viSNE expression heatmaps (concatenated files)

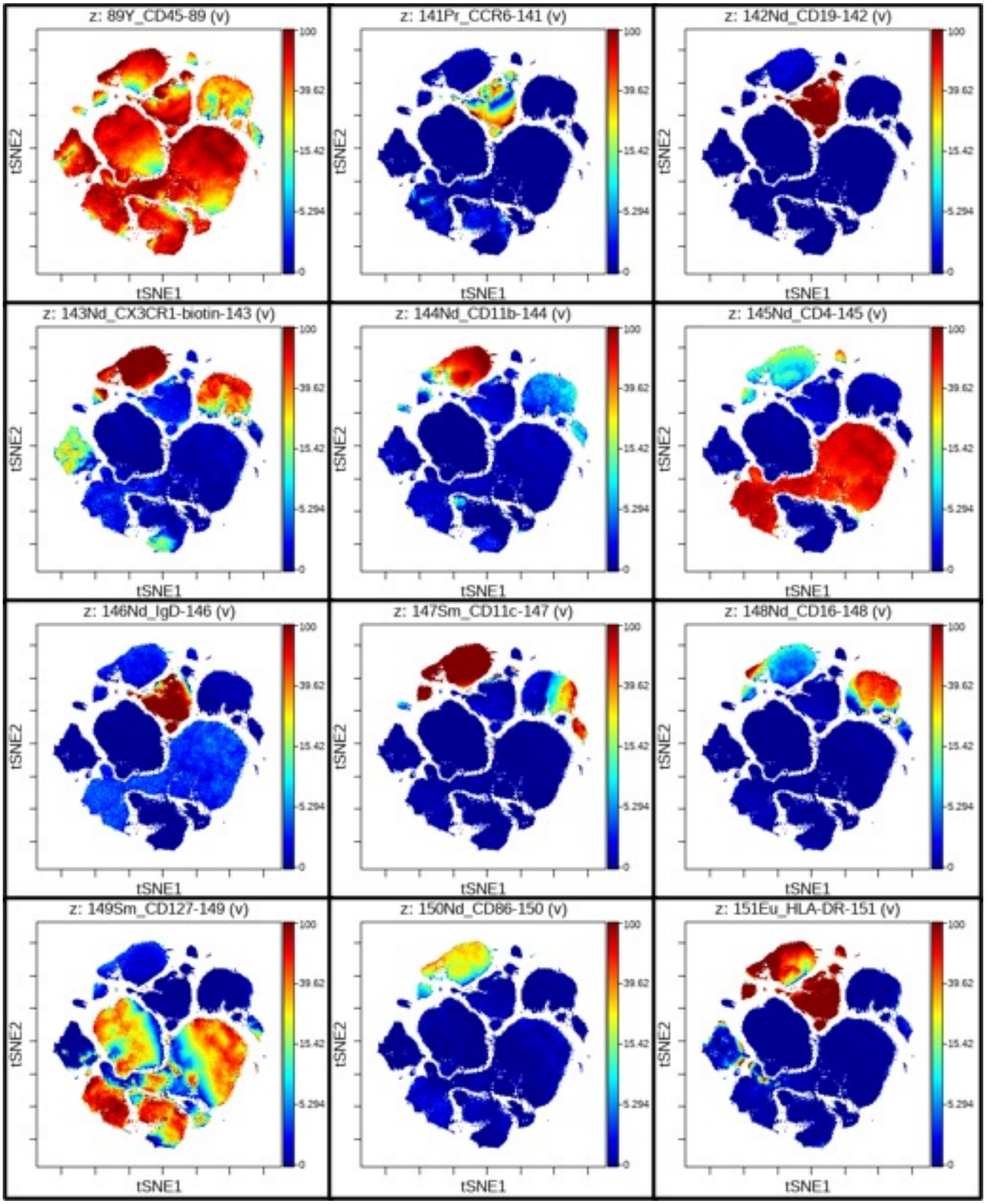

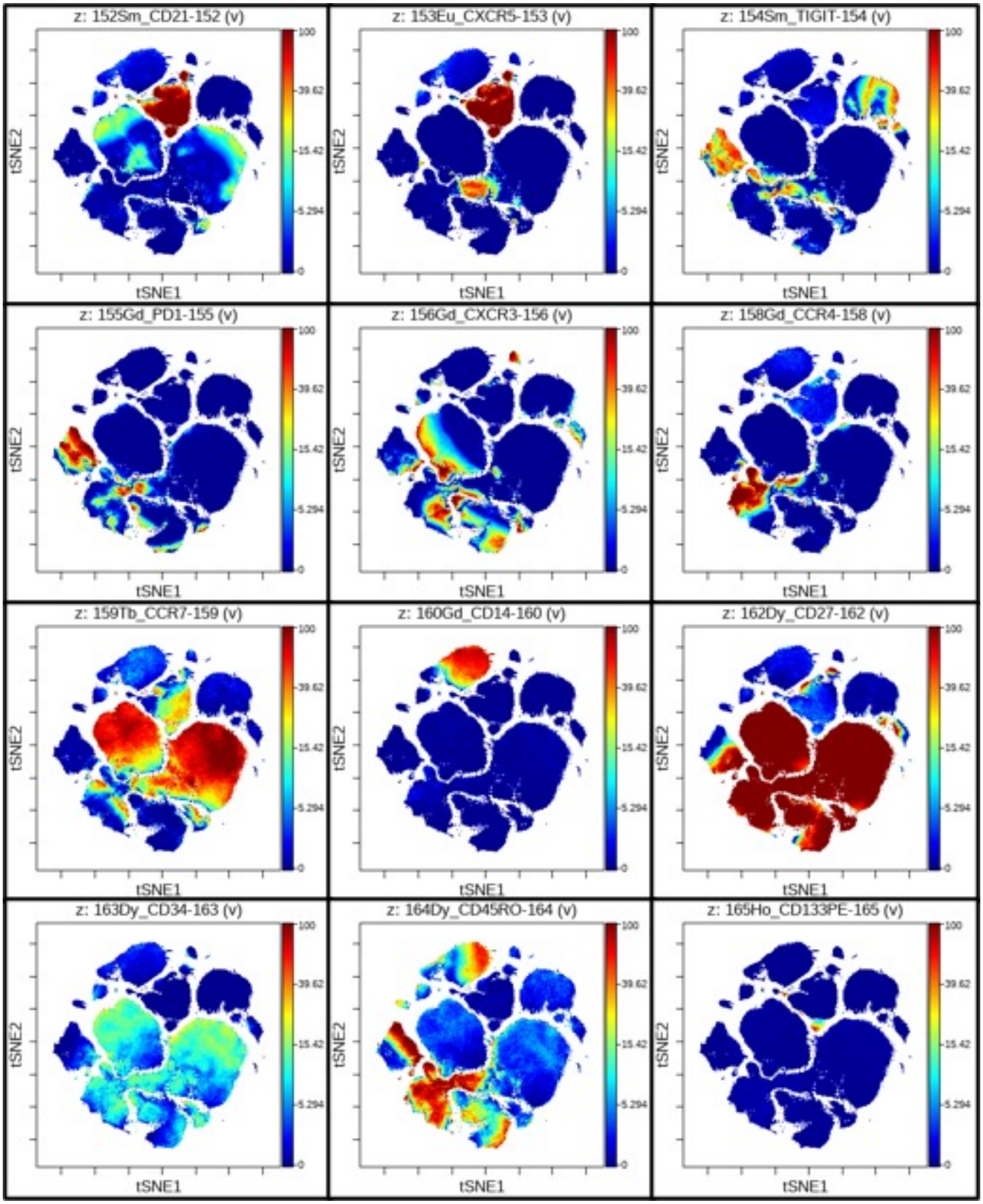
z: 152Sm_CD21-152 (v)
z: 153Eu_CXCR5-153 (v)
z: 154Sm_TIGIT-154 (v)
z: 155Gd_PD1-155 (v)
z: 156Gd_CXCR3-156 (v)
z: 158Gd_CCR4-158 (v)
z: 159Tb_CCR7-159 (v)
z: 160Gd_CD14-160 (v)
z: 162Dy_CD27-162 (v)
z: 163Dy_CD34-163 (v)
z: 164Dy_CD45RO-164 (v)
z: 165Ho_CD133PE-165 (v)
tSNE1
tSNE2
100
39.62
15.42
5.294
0

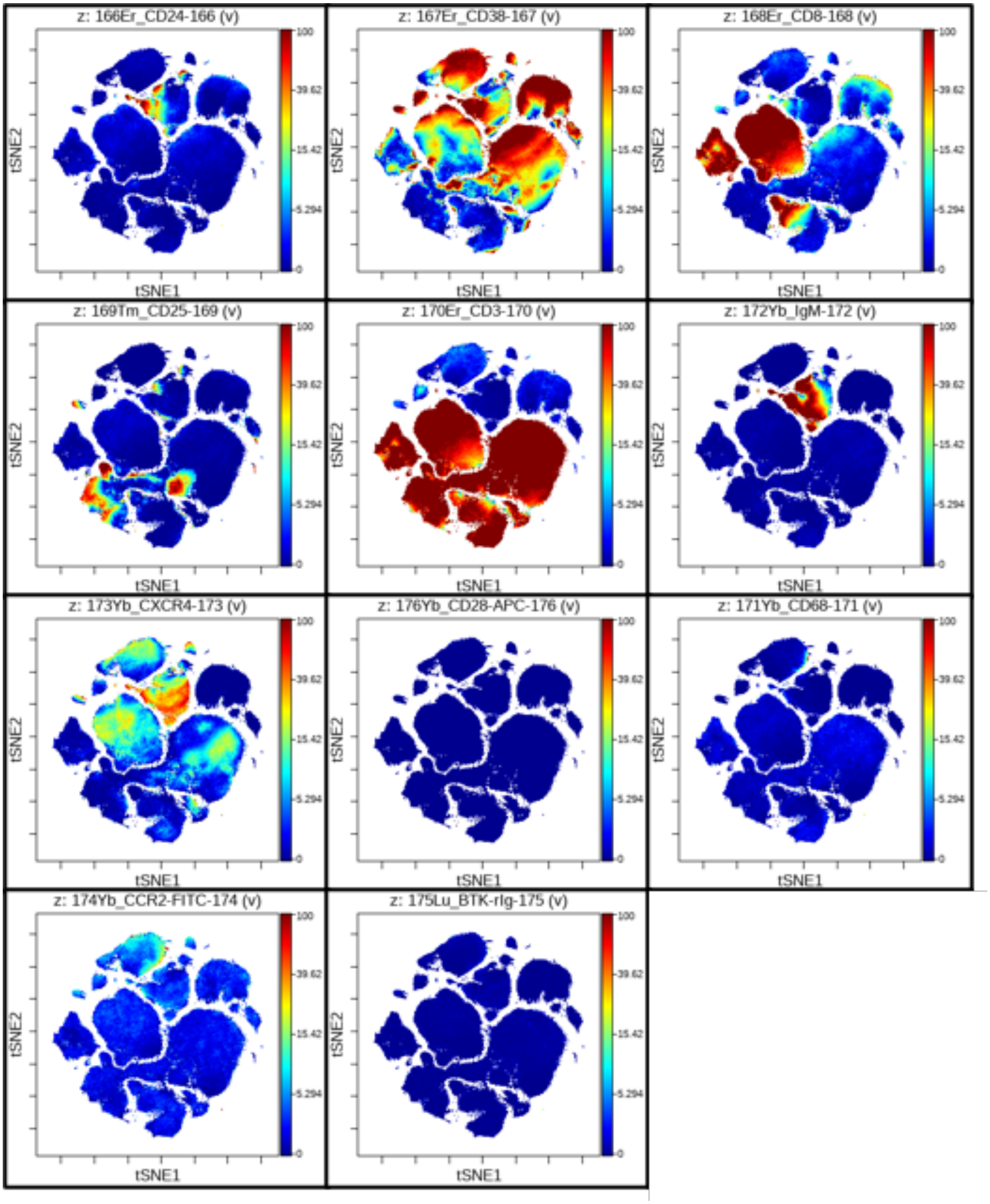
z: 166Er_CD24-166 (v)
z: 167Er_CD38-167 (v)
z: 168Er_CD8-168 (v)
z: 169Tm_CD25-169 (v)
z: 170Er_CD3-170 (v)
z: 172Yb_IgM-172 (v)
z: 173Yb_CXCR4-173 (v)
z: 176Yb_CD28-APC-176 (v)
z: 171Yb_CD68-171 (v)
z: 174Yb_CCR2-FITC-174 (v)
z: 175Lu_BTK-rIg-175 (v)
tSNE1
tSNE2
100
39.62
15.42
5.294
0

Figure S3. CyTOF assessment of human PBMCs suggest the activation of Innate immune responses in KCNK3-HPAH patients

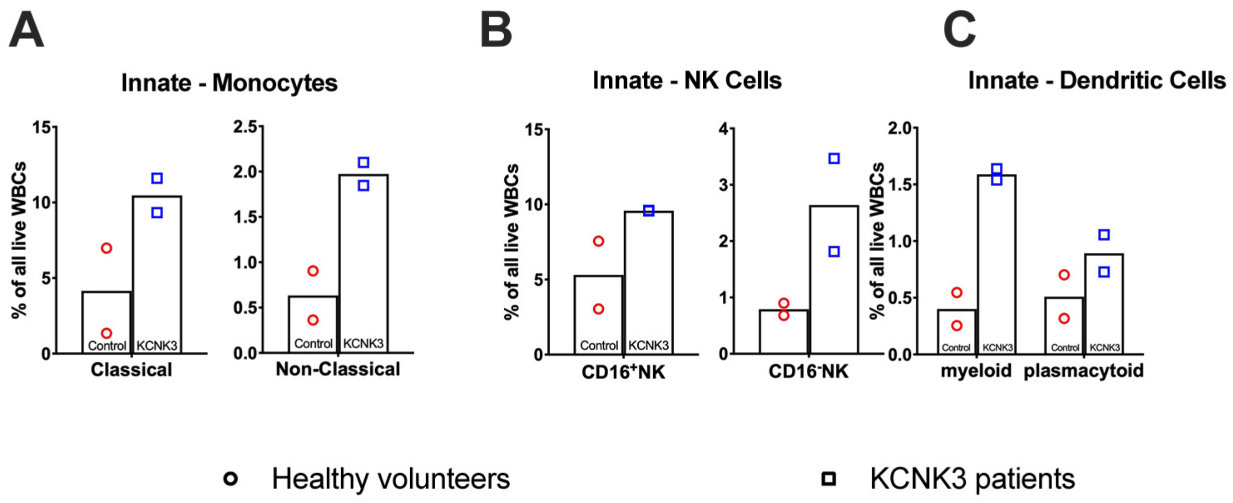

CyTOF assessment on human PBMCs demonstrated a 2-3x increase of innate immune components – classical and non-classical monocytes, $CD16^+$ and $CD16^-$ NK cells, and also dendritic cells (both myeloid and plasmacytoid) in KCNK3-HPAH patients.

Supplemental Table

Table S1. Gene Ontology of iPS Microarray

Probe Set ID	Gene Symbol	Control 1	Control 2	KCNK3 mutant	mRNA Accession
8147837	ZFPM2	3.753788	3.678257	6.14383	NM_012082
8116818	BMP6	3.767481	3.767481	6.720144	NM_001718
8102532	PDE5A	3.836234	3.836234	5.256213	NM_001083
8105267	ITGA2	3.869874	4.772655	6.09214	NM_002203
8151931	TSPYL5	3.871941	4.350015	6.135015	NM_033512
8132250	BMPER	3.906456	4.665087	6.831632	NM_133468
8154692	TEK	3.938014	4.757459	6.053513	NM_000459
8138689	SKAP2	3.95428	4.662279	6.095451	NM_001303468
8104758	NPR3	4.054159	4.269153	5.217735	NM_000908
7979241	BMP4	4.100423	4.100423	5.939707	NM_001202
8121850	HEY2	4.145161	3.968143	5.13365	NM_012259
7962212	PKP2	4.601914	5.21813	7.149449	NM_001005242
8091698	SHOX2	4.647913	4.647913	5.937673	NM_001163678
7970949	MAB21L1	4.853406	5.387149	3.272384	NM_005584
7923086	ASPM	4.943844	5.437356	3.84633	NM_001206846
7922976	PTGS2	5.08736	5.981365	3.949162	NM_000963
8155214	MELK	5.108953	5.253481	3.980186	NM_001256685
8097628	HHIP	5.276442	6.571479	8.17002	NM_022475
7929078	KIF20B	5.283451	5.621128	4.069167	NM_001284259
8151890	TP53INP1	5.398271	5.684606	6.72948	NM_001135733
8160441	CDKN2A	5.405371	6.286618	7.110746	NM_000077
8014591	HNF1B	5.409317	5.157039	4.133977	NM_000458
7979307	DLGAP5	5.446868	6.333407	4.598376	NM_001146015
8103728	HMGB2	5.505724	6.224369	4.825548	NM_001130688
8003322	CYBA	5.524947	5.353505	6.568742	NM_000101
8059376	SERPINE2	5.621711	6.705077	7.794783	NM_001136528
7901010	KIF2C	5.636137	5.622636	4.457448	NM_001297655

7926259	MCM10	5.736029	6.203403	4.849039	NM_018518
7909708	CENPF	5.79323	5.969531	4.298212	NM_016343
8144153	NCAPG2	5.795675	6.171447	4.87952	NM_001281932
8175217	GPC4	5.863088	5.970106	4.770126	NM_001448
7929032	FAS	5.89383	6.509405	7.874172	NM_000043
8001133	SHCBP1	5.92767	6.45394	5.09224	NM_024745
8110084	MSX2	6.030248	5.542302	4.6626	NM_002449
8123864	TFAP2A	6.066299	7.289571	4.622367	NM_001032280
7950555	LRRC32	6.104	7.3752	8.674616	NM_001128922
8147777	CTHRC1	6.108233	6.072557	7.119371	NM_001256099
8054580	BUB1	6.180406	6.921506	5.317943	NM_001278616
8017262	BRIP1	6.182948	6.46583	5.283368	NM_032043
8132118	AQP1	6.307012	7.359815	4.749716	NM_001185060
8007071	CDC6	6.339029	6.576197	4.692752	NM_001254
7999387	EMP2	6.341525	6.493083	4.880129	NM_001424
8021418	MALT1	6.405386	6.026776	5.133571	NM_006785
7985285	ARNT2	6.516064	5.323734	4.196272	NM_014862
7960340	FOXM1	6.610526	6.677169	5.407912	NM_001243088
8012403	AURKB	6.772769	7.014205	5.745514	NM_001256834
8015412	JUP	6.854382	7.575547	8.795095	NM_002230
7900699	CDC20	6.876464	7.557014	6.038435	NM_001255
7937020	MKI67	6.987939	7.336534	5.973728	NM_001145966
8141395	MCM7	7.124069	7.22803	6.039013	NM_001278595
8066822	SULF2	7.295147	7.221128	6.206872	NM_001161841
8039484	IL11	7.597742	6.657686	5.072037	NM_000641
8137526	INSIG1	7.694956	7.303686	5.959184	NM_005542
7977397	CRIP2	7.732655	7.478788	8.912133	NM_001270837
8160452	CDKN2B	7.976059	8.152885	9.333387	NM_004936
8131803	IL6	8.02247	8.191318	4.911787	NM_000600
8139488	IGFBP3	8.174747	8.573958	9.554642	NM_000598
7994109	PLK1	8.364383	8.736185	7.011639	NM_005030

8075310	LIF	8.520697	7.815692	6.476033	NM_001257135
7931097	HTRA1	8.542815	8.583393	9.595797	NM_002775
8152617	HAS2	8.588274	6.542852	4.092417	NM_005328
8163637	TNC	9.691027	8.838282	7.285433	NM_002160
7961514	MGP	2.687491	4.406413	7.548132	NM_000900
8120932	PRSS35	2.75527	3.319588	5.172153	NM_001170423
8094870	SHISA3	2.864613	4.569277	7.314581	NM_001080505
8122860	MYCT1	3.106201	3.392666	7.937538	NM_025107
7982008	SNORD115-1	3.174843	3.291023	5.064928	NR_001291
7982032	SNORD115-1	3.174843	3.291023	5.064928	NR_001291
7982038	SNORD115-1	3.174843	3.291023	5.064928	NR_001291
7982018	SNORD115-1	3.317688	3.206405	5.043474	NR_001291
8091306	PLSCR4	3.350403	3.474069	5.256557	NM_001128304
7982090	SNORD115-1	3.350403	3.22574	5.108373	NR_001291
8171921	DMD	3.370805	3.937013	5.350054	NM_000109
7965410	DCN	3.578554	3.405142	6.434369	NM_001920
7961365	MANSC1	3.592233	4.358784	6.209813	NM_018050
7982006	SNORD116-29	3.599633	3.173689	4.624912	NR_003360
8111255	CDH10	3.673683	3.562248	5.453468	NM_006727
8154135	SLC1A1	3.686099	4.6404	6.031627	NM_004170
8008819	YPEL2	3.686474	4.204033	5.071095	NM_001005404
7934997	PPP1R3C	3.71704	3.815421	5.075673	NM_005398
8071044	TEKT4P2	3.793409	3.324506	6.080981	NR_038327
8140668	SEMA3A	3.819222	4.367931	5.142897	NM_006080
8037251	PSG7	3.823463	3.746312	5.713368	NM_001206650
8177120	TEKT4P2	3.858858	3.986691	5.206972	NR_038327
7937039	EBF3	3.880946	4.354321	5.281522	NM_001005463
8103695	MFAP3L	3.892884	4.373666	5.170141	NM_001009554
7951372	CASP4	3.959921	4.428205	5.480414	NM_001225
8071051	TEKT4P2	3.97568	3.526222	6.014978	NR_038327
8057771	STAT4	3.983449	4.534959	5.855446	NM_001243835

8093166	PIGZ	4.019444	4.094553	5.116675	NM_025163
8058063	RFTN2	4.025315	4.223752	5.449033	NM_144629
7903227	PALMD	4.041473	4.088499	5.387241	NM_017734
7913593	TCEA3	4.041473	4.433648	5.252097	NM_003196
8132710	C7orf69	4.045653	4.65313	5.694642	NM_001302627
7969933	BIVM	4.086177	3.580841	5.031815	AK126520
8126853	PTCHD4	4.114315	4.278626	5.931394	NM_001013732
8104107	TRIML2	4.15382	4.188099	5.927236	NM_001303419
8144802	PDGFRL	4.160328	4.507992	6.426348	NM_006207
7939007	LUZP2	4.174322	3.863904	6.507897	NM_001009909
7976816	SNORD114-3	4.181602	2.264039	8.074939	NR_003195
7982084	SNORD115-1	4.185841	4.042267	6.850734	NR_001291
7926127	CELF2	4.235732	3.836234	5.121476	NM_001025076
8074606	USP41	4.24402	4.24402	5.304937	XM_006724407
7987405	RASGRP1	4.254426	4.443706	5.398895	NM_001128602
8151369	SBSPON	4.277519	5.68586	8.40913	NM_153225
8065537	LOC100134868	4.339894	4.718232	3.372141	NR_004846
8074168	LINC01296	4.340918	4.254585	5.370951	NR_122112
7977452	LINC01296	4.401941	4.416226	5.806	NR_122112
8084891	FAM43A	4.456332	5.632363	7.231817	NM_153690
8172425	SLC38A5	4.473644	3.962887	5.223432	NM_033518
8102200	DKK2	4.486034	3.882394	5.335907	NM_014421
7906264	PEAR1	4.557213	5.39544	6.429925	NM_001080471
8047784	ZDBF2	4.578517	5.402641	6.229595	NM_001285549
8019857	NDC80	4.579016	5.079308	3.714298	NM_006101
8084717	ST6GAL1	4.57989	5.216748	6.305926	NM_003032
8128565	POPDC3	4.664339	4.872491	3.60953	NM_022361
8088602	MAGI1	4.688179	5.045355	6.04172	NM_001033057
8045148	MZT2B	4.711442	4.548017	5.670253	XM_011511889
8055284	MZT2B	4.711442	4.548017	5.670253	XM_011511889
7942941	CCDC81	4.719901	4.791894	6.001727	NM_001156474

8005171	TRPV2	4.722057	4.853652	6.194136	NM_016113
8083494	MME	4.743834	4.606383	3.567095	NM_000902
7976073	FLRT2	4.752108	5.071802	6.184301	NM_013231
7985213	CHRNA5	4.764605	5.34329	3.763995	NM_000745
7927099	LINC00839	4.779306	4.602592	6.025829	NR_026827
8144786	SLC7A2	4.788666	6.418729	7.950139	NM_001008539
8144036	XRCC2	4.8424	5.219393	3.802933	NM_005431
8077731	FANCD2	4.846626	5.465368	4.144672	NM_001018115
7927606	PRKG1	4.848463	5.045356	6.173856	NM_001098512
7954065	GPRC5A	4.906563	5.374654	6.483192	NM_003979
8123637	HTATSF1P2	4.93336	5.702828	6.741109	NR_033884
8097973	GUCY1B3	4.947127	4.450735	3.488162	NM_000857
8145570	ESCO2	4.947631	5.476889	4.055732	NM_001017420
8146967	CRISPLD1	4.969331	4.969331	6.330464	NM_001286777
7920278	S100A3	4.971665	4.746975	6.290259	NM_002960
8037283	PSG4	5.010054	4.474876	8.656805	NM_001276495
8031646	ZNF667-AS1	5.012279	6.079345	3.427373	NR_036521
8020495	CABLES1	5.021638	5.345322	6.498011	NM_001100619
8058450	GPR1	5.104096	5.815599	6.761907	NM_001098199
7940530	MYRF	5.107993	5.834551	6.594298	NM_001127392
8034772	ASF1B	5.121846	5.580302	4.334764	NM_018154
8061483	LOC100134868	5.141119	5.096106	4.006495	NR_004846
8066925	PTGIS	5.171101	5.171101	7.882372	NM_000961
8151816	GEM	5.177461	5.415296	7.498481	NM_005261
8151871	CCNE2	5.17755	5.326646	3.836372	NM_057749
7981990	SNORD116-21	5.177966	4.575421	3.386732	NR_003335
7982663	PAK6	5.202819	5.345925	3.794493	NM_001128628
8145977	PLEKHA2	5.257662	5.939379	6.634385	NM_021623
8124391	HIST1H2AB	5.264209	6.099003	4.488202	NM_003513
8064894	LRRN4	5.282812	5.417805	4.233436	NM_152611
7940147	FAM111B	5.287727	5.748823	4.413286	NM_001142703

8013671	SPAG5	5.303306	5.474633	4.05334	NM_006461
8027247	ZNF93	5.320086	5.880729	4.342234	NM_031218
8099132	CYTL1	5.34528	5.350237	6.645666	NM_018659
7913566	HTR1D	5.352677	5.44017	6.508722	NM_000864
7957850	GAS2L3	5.379246	5.969814	4.37772	NM_001303130
7929258	KIF11	5.384888	5.8471	4.315806	NM_004523
7908204	HMCN1	5.441696	5.547084	6.766784	NM_031935
8109305	SYNPO	5.450247	5.527963	6.718337	NM_001109974
8092095	TNIK	5.492445	5.282734	3.520928	NM_001161560
8061247	RIN2	5.495635	6.138115	7.879323	NM_001242581
8073573	FAM109B	5.504136	5.580298	6.893072	NM_001002034
7927631	DKK1	5.540877	6.521642	8.028819	NM_012242
7899615	SERINC2	5.564854	6.225732	7.040218	NM_001199037
8097356	PLK4	5.590448	5.877633	4.230494	NM_001190799
8124166	MBOAT1	5.609929	5.224465	4.342823	NM_001080480
8073662	PARVB	5.632806	6.054708	6.962265	NM_001003828
7985873	TICRR	5.660335	5.780117	4.13255	NM_001308025
8155754	MAMDC2	5.670248	6.150896	7.149688	NM_153267
7982102	GABRA5	5.699346	6.632534	4.097627	NM_000810
7987192	SLC12A6	5.712003	6.071669	6.960524	NM_001042494
7970513	SKA3	5.765359	6.019273	4.47611	NM_001166017
8076757	CELSR1	5.787467	5.71789	4.588593	NM_014246
7976795	MEG3	5.797094	4.62799	8.763985	NR_002766
7991034	HOMER2	5.849521	5.078139	3.967296	NM_004839
8066136	RBL1	5.862245	6.185608	5.012794	NM_002895
8122426	PHACTR2	5.881479	6.045347	7.011969	NM_001100164
8035465	ISYNA1	5.902519	6.326935	7.378338	NM_001170938
8174201	BEX1	5.928147	6.508002	4.58607	NM_018476
8127932	TBX18	5.929869	6.054721	7.253736	NM_001080508
8124413	HIST1H4D	5.933147	5.453468	4.469857	NM_003539
8046895	FAM171B	5.955008	5.998316	7.253956	NM_177454

8122724	ULBP1	5.961882	5.959025	7.231815	NM_025218
7912520	NPPB	6.01553	6.068789	7.072328	NM_002521
7986214	SLCO3A1	6.047265	6.33946	3.611523	NM_001145044
8043602	NCAPH	6.080551	5.7222	4.645635	NM_001281710
8065353	THBD	6.083354	6.560971	8.489721	NM_000361
8122099	ENPP1	6.090173	5.905112	4.109865	NM_006208
8094301	SLIT2	6.135262	5.948711	5.038857	NM_001289135
7897449	SPSB1	6.137587	6.306324	7.571545	NM_025106
7971461	LCP1	6.146396	6.6564	4.214	NM_002298
8046695	ITGA4	6.150455	6.75944	7.62582	NM_000885
8134098	CDK14	6.160687	6.598107	7.430751	NM_001287135
7901993	CACHD1	6.18937	5.781844	4.523087	NM_001293274
8069252	PCBP3	6.198725	5.452059	4.406413	NM_001130141
7944955	PKNOX2	6.245763	6.03603	4.930992	NM_022062
7962375	PRICKLE1	6.278033	6.15418	4.402735	NM_001144881
7929334	CEP55	6.293085	6.581033	5.036497	NM_001127182
8012883	HS3ST3A1	6.298536	6.042541	4.932917	NM_006042
8040223	RRM2	6.300907	6.786909	5.11375	NM_001034
8021946	COLEC12	6.321092	5.90718	4.993235	NM_130386
8014974	TOP2A	6.327249	6.854273	4.822553	NM_001067
7981964	SNORD116-3	6.339323	6.179147	7.393049	NR_003318
7901460	GPX7	6.370981	6.580451	7.608717	NM_015696
8122637	SASH1	6.384047	7.24869	8.742763	NM_015278
8022692	DSC3	6.44559	6.065755	8.300377	NM_001941
8108301	KIF20A	6.50636	6.989044	5.540739	NM_005733
8005449	KRT17P2	6.506844	7.437622	4.950638	AK296148
8156199	DAPK1	6.583598	6.21024	4.459075	NM_001288729
8124527	HIST1H1B	6.618125	7.122617	5.781844	NM_005322
7999754	XYLT1	6.652751	6.496768	4.119151	NM_022166
8094278	NCAPG	6.672259	6.949751	5.391763	NM_022346
8033818	OLFM2	6.678961	7.120168	5.804385	NM_001304347

7902441	ST6GALNAC5	6.686492	7.688751	5.639627	NM_030965
7964602	LRIG3	6.689498	5.855251	5.065257	NM_001136051
8060897	PLCB4	6.692489	6.958646	5.807831	NM_000933
7982358	ARHGAP11A	6.714486	6.679373	5.076457	NM_001286479
8104022	PDLIM3	6.723104	7.247554	8.323392	NM_001114107
8166049	PRPS2	6.723617	7.484012	5.661592	NM_001039091
8075637	LARGE	6.735202	6.120577	4.951985	NM_004737
7899627	TINAGL1	6.764538	7.867495	9.416409	NM_001204414
8068671	BACE2	6.770382	6.915091	5.451259	NM_012105
7980908	FBLN5	6.811926	6.405494	7.963328	NM_006329
7909568	DTL	6.814297	7.144142	5.125301	NM_001286229
8124437	HIST1H3F	6.837243	6.804944	5.524141	NM_021018
8005097	HS3ST3B1	6.848228	6.542455	4.308916	NM_006041
8136248	MEST	6.851563	7.921887	9.269516	NM_001253900
8113726	PPIC	6.88954	6.961374	7.931062	NM_000943
8142981	PODXL	6.915294	8.047222	9.431723	NM_001018111
7938364	WEE1	7.017804	6.910364	5.440882	ENST00000524549
8071671	GNAZ	7.019323	6.80997	5.117898	NM_002073
8171297	MID1	7.03113	6.817859	5.862845	NM_000381
8172035	DYNLT3	7.072733	6.993057	8.060137	NM_006520
8086517	CDCP1	7.109221	6.308784	5.001067	NM_022842
7936494	GFRA1	7.119196	7.759834	5.153673	NM_001145453
7904158	OLFML3	7.150547	6.427267	5.400223	NM_001286352
8060813	MCM8	7.167406	7.469702	6.213185	NM_001281520
8098204	CPE	7.180418	7.107944	5.306978	NM_001873
8097098	USP53	7.198789	7.094873	8.274392	NM_019050
7990054	UACA	7.211989	7.884422	8.728663	NM_001008224
8131600	TSPAN13	7.272326	8.111202	8.880699	NM_014399
8153021	ST3GAL1	7.311047	7.733921	5.902519	NM_003033
8124388	HIST1H3B	7.325696	7.368342	6.000334	NM_003537
8046333	CYBRD1	7.331817	8.274347	9.861664	NM_001127383

8072687	MCM5	7.348068	7.269277	6.160971	NM_006739
8001784	CDH8	7.349384	7.907311	5.138951	NM_001796
7950307	UCP2	7.360543	6.714551	5.876097	NM_003355
8008784	PRR11	7.372391	7.340515	6.009403	NM_018304
8175871	L1CAM	7.405349	6.392854	4.482969	NM_000425
8030383	RCN3	7.444001	8.160577	9.005676	NM_020650
7951662	CRYAB	7.492001	7.712568	9.261177	NM_001289807
7901969	ROR1	7.508128	7.837547	8.7986	NM_001083592
8118890	SCUBE3	7.556987	7.424299	9.089632	NM_001303136
7932082	CCDC3	7.611732	5.931174	4.199731	NM_001282658
8138888	PDE1C	7.619255	7.007731	5.71167	NM_001191056
7956046	DGKA	7.682218	8.047695	8.974467	NM_001345
8001449	IRX3	7.686186	6.763125	5.903809	NM_024336
8116418	GFPT2	7.722575	7.365243	6.056204	NM_005110
7999468	LITAF	7.774823	8.401083	5.481526	NM_001136472
8108683	PCDHB2	7.826665	7.642568	5.407521	NM_018936
8015387	KRT17	7.879084	8.692177	4.921796	NM_000422
8115851	STC2	7.938939	8.277897	9.19849	NM_003714
8059854	ARL4C	8.012944	7.043461	6.047235	NM_001282431
8161701	TMEM2	8.033732	7.542506	6.329821	NM_001135820
7990345	SEMA7A	8.048428	7.646872	6.653291	NM_001146029
8054439	ST6GAL2	8.056755	7.422904	6.671877	NM_001142351
7930980	PLPP4	8.117237	6.940271	5.186088	NM_001030059
7948229	SLC43A3	8.155936	7.825219	5.539972	NM_001278201
8013042	KRT17P2	8.177467	9.178667	7.110447	AK296148
8117426	HIST1H2BH	8.182202	8.207098	6.600335	NM_003524
7960919	MFAP5	8.197567	8.670865	10.45437	NM_001297709
8001197	NETO2	8.232244	7.581448	6.031626	NM_001201477
8059905	COL6A3	8.280857	9.34433	7.145096	NM_004369
8170119	FHL1	8.357913	9.238449	10.05693	NM_001159699
8006433	CCL2	8.3782	9.556733	6.362932	NM_002982

7928589	PPIF	8.491174	8.482619	7.346016	NM_005729
7984540	KIF23	8.538855	8.299293	7.300292	NM_001281301
7997642	CRISPLD2	8.578808	7.69061	3.878607	NM_031476
8117368	HIST1H4C	8.675164	9.000552	7.755011	NM_003542
8109752	TENM2	8.681618	8.2613	5.533885	NM_001080428
7955637	KRT18	9.106567	9.797234	10.46973	NM_000224
7952268	THY1	9.24589	9.657022	7.613053	NM_001311160
8169240	PRPS1	9.43181	9.541888	7.767011	NM_001204402
8104461	---	4.412413	3.94606	5.30721	NONHSAT100420
7998927	---	4.714522	5.183132	3.620875	uc021tbe.1
7930380	ADD3	5.126945	6.023119	7.075558	NM_001121
8034783	ADGRL1	5.232126	5.685818	6.480236	NM_001008701
8158771	AIF1L	5.47957	5.280882	3.963605	NM_001185095
7902565	ADGRL2	5.62748	5.822192	7.070981	NM_001297704
8040362	---	5.632806	5.658631	6.66814	GENSCAN00000010282
8037387	---	5.638609	6.119893	4.730884	NONHSAT066617
7981317	---	5.715791	5.478179	8.456131	NONHSAT039752
8050766	ADCY3	5.776019	6.164147	4.92509	NM_004036
8154491	ADAMTSL1	5.802879	6.186495	4.232107	NM_001040272
7981514	AHNAK2	5.991465	5.931174	6.994463	NM_138420
8112327	---	6.559037	6.855026	5.681139	ENST00000497342
7985786	ACAN	6.699649	6.164265	4.538263	NM_001135
8171248	ANOS1	7.662767	8.269506	9.28553	NM_000216
7896752	---	8.069938	8.450727	4.518978	ENST00000387421
8165667	---	8.069938	8.450727	4.518978	ENST00000387421
8043375	---	8.316909	8.62455	4.255461	ENST00000387421
8132318	ANLN	8.681654	8.558188	7.171162	NM_001284301

Table S2. RNA-Seq gene expression in Whole Mouse Lung

ensembl_ gene_id	mgi_ symbol	Description	Control	Control	KCNK3$^{fl/fl}$	KCNK3$^{fl/fl}$	Fold Change
ENSMUSG00000018381	Abi3	ABI gene family, member 3 [Source:MGI Symbol;Acc:MGI:1913860]	11.2	11.2	11.5	11.6	1.3
ENSMUSG00000044337	Ackr3	atypical chemokine receptor 3 [Source:MGI Symbol;Acc:MGI:109562]	12.3	12.2	11.8	11.7	-1.4
ENSMUSG00000017307	Acot8	acyl-CoA thioesterase 8 [Source:MGI Symbol;Acc:MGI:2158201]	8.9	9.0	9.3	9.2	1.3
ENSMUSG00000058145	Adamts17	a disintegrin-like and metallopeptidase (reprolysin type) with thrombospondin type 1 motif, 17 [Source:MGI Symbol;Acc:MGI:3588195]	8.8	8.8	9.2	9.2	1.3
ENSMUSG00000031448	Adprhl1	ADP-ribosylhydrolase like 1 [Source:MGI Symbol;Acc:MGI:2442168]	5.2	5.4	6.3	6.2	2.0
ENSMUSG00000058620	Adra2b	adrenergic receptor, alpha 2b [Source:MGI Symbol;Acc:MGI:87935]	5.3	5.2	4.7	4.6	-1.5
ENSMUSG00000010601	Apol7a	apolipoprotein L 7a [Source:MGI Symbol;Acc:MGI:1923011]	5.3	5.4	6.0	5.9	1.5
ENSMUSG00000028919	Arhgef19	Rho guanine nucleotide exchange factor (GEF) 19 [Source:MGI Symbol;Acc:MGI:1925912]	9.6	9.6	9.9	10.0	1.3
ENSMUSG00000047446	Arl4a	ADP-ribosylation factor-like 4A [Source:MGI Symbol;Acc:MGI:99437]	11.4	11.3	11.0	10.9	-1.3
ENSMUSG00000037053	Azgp1	alpha-2-glycoprotein 1, zinc [Source:MGI Symbol;Acc:MGI:103163]	7.7	7.7	8.2	8.1	1.3
ENSMUSG00000021070	Bdkrb2	bradykinin receptor, beta 2 [Source:MGI Symbol;Acc:MGI:102845]	7.9	7.9	7.4	7.6	-1.4
ENSMUSG00000038871	Bpgm	2,3-bisphosphoglycerate mutase [Source:MGI Symbol;Acc:MGI:1098242]	11.1	11.3	10.7	10.7	-1.5
ENSMUSG00000063130	Calml3	calmodulin-like 3 [Source:MGI Symbol;Acc:MGI:1917655]	7.4	7.3	6.9	7.1	-1.3
ENSMUSG00000031883	Car7	carbonic anhydrase 7 [Source:MGI Symbol;Acc:MGI:103100]	6.4	6.5	6.0	6.0	-1.3
ENSMUSG00000031143	Ccdc22	coiled-coil domain containing 22 [Source:MGI Symbol;Acc:MGI:1859608]	4.2	3.9	5.0	5.3	2.1
ENSMUSG00000042262	Ccr8	chemokine (C-C motif) receptor 8 [Source:MGI Symbol;Acc:MGI:1201402]	5.9	6.0	5.4	5.5	-1.4

ENSMUSG00000069607	Cd300lh	CD300 antigen like family member H [Source:MGI Symbol;Acc:MGI:2687214]	3.6	3.6	4.3	4.3	1.6
ENSMUSG00000030798	Cd37	CD37 antigen [Source:MGI Symbol;Acc:MGI:88330]	7.0	6.9	6.5	6.6	-1.4
ENSMUSG00000027435	Cd93	CD93 antigen [Source:MGI Symbol;Acc:MGI:106664]	15.1	15.1	15.5	15.4	1.3
ENSMUSG00000021175	Cdca7l	cell division cycle associated 7 like [Source:MGI Symbol;Acc:MGI:2384982]	8.5	8.4	8.1	8.0	-1.3
ENSMUSG00000000305	Cdh4	cadherin 4 [Source:MGI Symbol;Acc:MGI:99218]	9.8	9.8	9.3	9.3	-1.4
ENSMUSG00000002835	Chaf1a	chromatin assembly factor 1, subunit A (p150) [Source:MGI Symbol;Acc:MGI:1351331]	9.3	9.2	8.7	8.8	-1.4
ENSMUSG00000022945	Chaf1b	chromatin assembly factor 1, subunit B (p60) [Source:MGI Symbol;Acc:MGI:1314881]	8.2	8.1	7.8	7.8	-1.3
ENSMUSG00000030365	Clec2i	C-type lectin domain family 2, member i [Source:MGI Symbol;Acc:MGI:2136650]	7.4	7.4	7.0	7.1	-1.3
ENSMUSG00000025064	Col17a1	collagen, type XVII, alpha 1 [Source:MGI Symbol;Acc:MGI:88450]	8.5	8.5	8.9	8.8	1.3
ENSMUSG00000018916	Csf2	colony stimulating factor 2 (granulocyte-macrophage) [Source:MGI Symbol;Acc:MGI:1339752]	6.5	6.6	7.2	7.2	1.6
ENSMUSG00000052336	Cx3cr1	chemokine (C-X3-C motif) receptor 1 [Source:MGI Symbol;Acc:MGI:1333815]	9.8	9.8	10.3	10.3	1.4
ENSMUSG00000027983	Cyp2u1	cytochrome P450, family 2, subfamily u, polypeptide 1 [Source:MGI Symbol;Acc:MGI:1918769]	7.3	7.3	7.7	7.6	1.3
ENSMUSG00000047642	D930020B18Rik	RIKEN cDNA D930020B18 gene [Source:MGI Symbol;Acc:MGI:2442001]	6.4	6.3	7.1	6.9	1.6
ENSMUSG00000036862	Dchs1	dachsous 1 (Drosophila) [Source:MGI Symbol;Acc:MGI:2685011]	11.4	11.5	11.8	11.8	1.3
ENSMUSG00000043051	Disc1	disrupted in schizophrenia 1 [Source:MGI Symbol;Acc:MGI:2447658]	7.2	7.1	7.7	7.5	1.3
ENSMUSG00000047517	Dmbt1	deleted in malignant brain tumors 1 [Source:MGI Symbol;Acc:MGI:106210]	3.1	3.0	3.7	3.7	1.6
ENSMUSG00000036766	Dner	delta/notch-like EGF repeat containing [Source:MGI Symbol;Acc:MGI:2152889]	6.3	6.4	6.0	5.9	-1.3
ENSMUSG00000040631	Dok4	docking protein 4 [Source:MGI Symbol;Acc:MGI:2148865]	11.0	11.0	11.3	11.4	1.3

ENSMUSG00000078580	E430018J23Rik	RIKEN cDNA E430018J23 gene [Source:MGI Symbol;Acc:MGI:2141981]	8.7	8.7	9.2	9.0	1.3
ENSMUSG00000010476	Ebf3	early B cell factor 3 [Source:MGI Symbol;Acc:MGI:894289]	5.0	5.1	4.4	4.5	-1.5
ENSMUSG00000022122	Ednrb	endothelin receptor type B [Source:MGI Symbol;Acc:MGI:102720]	11.6	11.5	11.8	12.0	1.3
ENSMUSG00000022203	Efs	embryonal Fyn-associated substrate [Source:MGI Symbol;Acc:MGI:105311]	8.8	8.8	9.2	9.4	1.4
ENSMUSG00000019989	Enpp3	ectonucleotide pyrophosphatase/phosphodiesterase 3 [Source:MGI Symbol;Acc:MGI:2143702]	7.5	7.3	7.0	7.0	-1.3
ENSMUSG00000028644	Ermap	erythroblast membrane-associated protein [Source:MGI Symbol;Acc:MGI:1349816]	5.8	6.2	4.9	5.0	-2.1
ENSMUSG00000019768	Esr1	estrogen receptor 1 (alpha) [Source:MGI Symbol;Acc:MGI:1352467]	7.7	7.6	8.9	8.9	2.3
ENSMUSG00000019909	Fam162b	family with sequence similarity 162, member B [Source:MGI Symbol;Acc:MGI:1924546]	6.9	7.2	7.9	8.0	1.8
ENSMUSG00000042788	Fam166b	family with sequence similarity 166, member B [Source:MGI Symbol;Acc:MGI:2445194]	7.6	7.5	7.9	7.9	1.3
ENSMUSG00000050493	Fam167b	family with sequence similarity 167, member B [Source:MGI Symbol;Acc:MGI:2668032]	6.4	6.3	6.0	5.9	-1.3
ENSMUSG00000073805	Fam196a	family with sequence similarity 196, member A [Source:MGI Symbol;Acc:MGI:3605068]	7.0	7.0	6.3	6.2	-1.7
ENSMUSG00000034532	Fbxo16	F-box protein 16 [Source:MGI Symbol;Acc:MGI:1354706]	6.1	6.0	6.7	6.5	1.5
ENSMUSG00000089984	Fbxo24	F-box protein 24 [Source:MGI Symbol;Acc:MGI:1918426]	5.8	5.7	5.3	5.3	-1.4
ENSMUSG00000090173	Fbxw10	F-box and WD-40 domain protein 10 [Source:MGI Symbol;Acc:MGI:3052463]	6.4	6.3	5.6	5.6	-1.7
ENSMUSG00000037106	Fer1l6	fer-1-like 6 (C. elegans) [Source:MGI Symbol;Acc:MGI:3645398]	6.4	6.4	5.4	5.4	-1.9
ENSMUSG00000044453	Ffar1	free fatty acid receptor 1 [Source:MGI Symbol;Acc:MGI:2684079]	6.3	6.4	5.6	5.6	-1.7
ENSMUSG00000047787	Flrt1	fibronectin leucine rich transmembrane protein 1 [Source:MGI Symbol;Acc:MGI:3026647]	6.0	5.9	5.6	5.6	-1.3
ENSMUSG00000029212	Gabrb1	gamma-aminobutyric acid (GABA) A receptor, subunit beta 1 [Source:MGI Symbol;Acc:MGI:95619]	5.5	5.7	6.2	6.1	1.4

ENSMUSG00000028671	Gale	galactose-4-epimerase, UDP [Source:MGI Symbol;Acc:MGI:1921496]	8.8	8.8	9.2	9.1	1.3
ENSMUSG00000015944	Gatsl2	GATS protein-like 2 [Source:MGI Symbol;Acc:MGI:1933384]	11.4	11.5	11.8	11.8	1.3
ENSMUSG00000038508	Gdf15	growth differentiation factor 15 [Source:MGI Symbol;Acc:MGI:1346047]	6.9	6.9	7.4	7.3	1.4
ENSMUSG00000061104	Gm10094	predicted gene 10094 [Source:MGI Symbol;Acc:MGI:3704317]	6.2	6.1	5.8	5.7	-1.3
ENSMUSG00000091556	Gm14569	predicted gene 14569 [Source:MGI Symbol;Acc:MGI:3705243]	6.0	5.9	6.4	6.5	1.5
ENSMUSG00000031748	Gnao1	guanine nucleotide binding protein, alpha O [Source:MGI Symbol;Acc:MGI:95775]	10.3	10.2	10.7	10.9	1.4
ENSMUSG00000015812	Gnrh1	gonadotropin releasing hormone 1 [Source:MGI Symbol;Acc:MGI:95789]	6.1	5.7	5.1	5.2	-1.7
ENSMUSG00000039720	Got1l1	glutamic-oxaloacetic transaminase 1-like 1 [Source:MGI Symbol;Acc:MGI:1923865]	4.6	4.7	5.4	5.3	1.6
ENSMUSG00000031700	Gpt2	glutamic pyruvate transaminase (alanine aminotransferase) 2 [Source:MGI Symbol;Acc:MGI:1915391]	9.3	9.4	9.8	9.7	1.3
ENSMUSG00000046182	Gsg1l	GSG1-like [Source:MGI Symbol;Acc:MGI:2685483]	8.2	8.1	7.5	7.6	-1.5
ENSMUSG00000023982	Guca1a	guanylate cyclase activator 1a (retina) [Source:MGI Symbol;Acc:MGI:102770]	5.6	6.1	4.6	4.7	-2.4
ENSMUSG00000051839	Gypa	glycophorin A [Source:MGI Symbol;Acc:MGI:95880]	6.9	7.4	4.5	3.7	-8.3
ENSMUSG00000038422	Hdhd3	haloacid dehalogenase-like hydrolase domain containing 3 [Source:MGI Symbol;Acc:MGI:1919998]	7.4	7.5	7.8	7.9	1.3
ENSMUSG00000041180	Hectd2	HECT domain containing 2 [Source:MGI Symbol;Acc:MGI:2442663]	8.2	8.3	8.0	7.8	-1.3
ENSMUSG00000043342	Hoxd9	homeobox D9 [Source:MGI Symbol;Acc:MGI:96210]	6.5	6.2	5.3	5.5	-1.9
ENSMUSG00000029096	Htra3	HtrA serine peptidase 3 [Source:MGI Symbol;Acc:MGI:1925808]	10.2	10.2	10.6	10.8	1.4
ENSMUSG00000079017	Ifi27l2a	interferon, alpha-inducible protein 27 like 2A [Source:MGI Symbol;Acc:MGI:1924183]	9.4	9.5	10.0	10.0	1.4
ENSMUSG00000031111	Igsf1	immunoglobulin superfamily, member 1 [Source:MGI Symbol;Acc:MGI:2147913]	4.7	4.7	5.4	5.3	1.6

ENSMUSG00000049093	Il23r	interleukin 23 receptor [Source:MGI Symbol;Acc:MGI:2181693]	5.6	5.6	4.8	5.0	-1.6
ENSMUSG00000068227	Il2rb	interleukin 2 receptor, beta chain [Source:MGI Symbol;Acc:MGI:96550]	9.9	9.8	9.4	9.3	-1.4
ENSMUSG00000037801	Iqch	IQ motif containing H [Source:MGI Symbol;Acc:MGI:1925500]	4.6	4.4	5.5	5.1	1.8
ENSMUSG00000018470	Kcnab3	potassium voltage-gated channel, shaker-related subfamily, beta member 3 [Source:MGI Symbol;Acc:MGI:1336208]	6.7	6.6	5.9	5.5	-1.9
ENSMUSG00000025597	Klhl4	kelch-like 4 [Source:MGI Symbol;Acc:MGI:2442829]	8.2	8.2	7.8	7.6	-1.4
ENSMUSG00000030167	Klrc1	killer cell lectin-like receptor subfamily C, member 1 [Source:MGI Symbol;Acc:MGI:1336161]	6.1	6.1	5.6	5.6	-1.4
ENSMUSG00000100916	Lhb	luteinizing hormone beta [Source:MGI Symbol;Acc:MGI:96782]	7.1	6.9	7.6	7.5	1.5
ENSMUSG00000030427	Lilra6	leukocyte immunoglobulin-like receptor, subfamily A (with TM domain), member 6 [Source:MGI Symbol;Acc:MGI:1195969]	7.9	7.7	8.2	8.3	1.4
ENSMUSG00000022595	Lypd2	Ly6/Plaur domain containing 2 [Source:MGI Symbol;Acc:MGI:1915561]	8.9	8.7	9.2	9.2	1.3
ENSMUSG00000025963	Mdh1b	malate dehydrogenase 1B, NAD (soluble) [Source:MGI Symbol;Acc:MGI:1923918]	7.1	7.1	7.5	7.5	1.3
ENSMUSG00000051855	Mest	mesoderm specific transcript [Source:MGI Symbol;Acc:MGI:96968]	9.2	9.2	8.8	8.7	-1.3
ENSMUSG00000054641	Mmrn1	multimerin 1 [Source:MGI Symbol;Acc:MGI:1918195]	12.7	12.6	12.2	12.3	-1.3
ENSMUSG00000079429	Mroh2a	maestro heat-like repeat family member 2A [Source:MGI Symbol;Acc:MGI:3705228]	5.9	5.8	5.4	5.4	-1.3
ENSMUSG00000025141	Myadml2	myeloid-associated differentiation marker-like 2 [Source:MGI Symbol;Acc:MGI:1915765]	3.3	3.0	4.9	4.9	3.4
ENSMUSG00000022346	Myc	myelocytomatosis oncogene [Source:MGI Symbol;Acc:MGI:97250]	9.5	9.4	8.9	8.8	-1.5
ENSMUSG00000041361	Myzap	myocardial zonula adherens protein [Source:MGI Symbol;Acc:MGI:2142908]	10.7	10.7	11.2	11.2	1.4
ENSMUSG00000030380	Mzf1	myeloid zinc finger 1 [Source:MGI Symbol;Acc:MGI:107457]	6.3	6.3	6.8	6.7	1.4

ENSMUSG00000006471	Ndor1	NADPH dependent diflavin oxidoreductase 1 [Source:MGI Symbol;Acc:MGI:1926047]	11.1	11.0	10.5	10.5	-1.5
ENSMUSG00000031478	Nek3	NIMA (never in mitosis gene a)-related expressed kinase 3 [Source:MGI Symbol;Acc:MGI:1344371]	7.5	7.4	7.8	7.9	1.3
ENSMUSG00000029832	Nfe2l3	nuclear factor, erythroid derived 2, like 3 [Source:MGI Symbol;Acc:MGI:1339958]	7.6	7.6	7.3	7.2	-1.3
ENSMUSG00000015468	Notch4	notch 4 [Source:MGI Symbol;Acc:MGI:107471]	13.2	13.4	13.7	13.8	1.4
ENSMUSG00000059791	Nrm	nurim (nuclear envelope membrane protein) [Source:MGI Symbol;Acc:MGI:2146855]	9.0	8.9	8.6	8.6	-1.3
ENSMUSG00000020297	Nsg2	neuron specific gene family member 2 [Source:MGI Symbol;Acc:MGI:1202070]	7.3	7.2	7.0	6.9	-1.3
ENSMUSG00000045392	Olfr1033	olfactory receptor 1033 [Source:MGI Symbol;Acc:MGI:3030867]	6.2	6.4	6.8	6.8	1.4
ENSMUSG00000037366	Pafah2	platelet-activating factor acetylhydrolase 2 [Source:MGI Symbol;Acc:MGI:2140321]	10.4	10.3	10.7	10.7	1.3
ENSMUSG00000030513	Pcsk6	proprotein convertase subtilisin/kexin type 6 [Source:MGI Symbol;Acc:MGI:102897]	10.6	10.6	10.3	10.1	-1.3
ENSMUSG00000035246	Pcyt1b	phosphate cytidylyltransferase 1, choline, beta isoform [Source:MGI Symbol;Acc:MGI:2147987]	7.7	7.7	7.3	7.4	-1.3
ENSMUSG00000050751	Pgbd5	piggyBac transposable element derived 5 [Source:MGI Symbol;Acc:MGI:2429955]	5.1	5.1	4.6	4.6	-1.4
ENSMUSG00000041202	Pla2g2d	phospholipase A2, group IID [Source:MGI Symbol;Acc:MGI:1341796]	6.6	6.7	6.2	6.3	-1.3
ENSMUSG00000026173	Plcd4	phospholipase C, delta 4 [Source:MGI Symbol;Acc:MGI:107469]	6.1	6.3	6.8	6.7	1.5
ENSMUSG00000044667	Plppr4	phospholipid phosphatase related 4 [Source:MGI Symbol;Acc:MGI:106530]	4.7	4.7	5.2	5.3	1.4
ENSMUSG00000070802	Pnmal2	PNMA-like 2 [Source:MGI Symbol;Acc:MGI:3645856]	8.9	9.0	9.2	9.3	1.3
ENSMUSG00000020974	Pole2	polymerase (DNA directed), epsilon 2 (p59 subunit) [Source:MGI Symbol;Acc:MGI:1197514]	7.5	7.4	7.0	7.1	-1.4
ENSMUSG00000061718	Ppp1r1b	protein phosphatase 1, regulatory (inhibitor) subunit 1B [Source:MGI Symbol;Acc:MGI:94860]	9.2	9.3	9.8	9.8	1.4
ENSMUSG00000038151	Prdm1	PR domain containing 1, with ZNF domain [Source:MGI Symbol;Acc:MGI:99655]	9.6	9.5	9.8	9.9	1.3

ENSMUSG00000010175	Prox1	prospero homeobox 1 [Source:MGI Symbol;Acc:MGI:97772]	10.3	10.3	9.9	10.0	-1.3
ENSMUSG00000053198	Prx	periaxin [Source:MGI Symbol;Acc:MGI:108176]	14.2	14.2	14.5	14.6	1.3
ENSMUSG00000097487	Ptges3l	prostaglandin E synthase 3 (cytosolic)-like [Source:MGI Symbol;Acc:MGI:1916146]	5.2	5.3	5.5	5.6	1.3
ENSMUSG00000038751	Ptk6	PTK6 protein tyrosine kinase 6 [Source:MGI Symbol;Acc:MGI:99683]	5.8	6.0	6.9	6.8	1.9
ENSMUSG00000026204	Ptprn	protein tyrosine phosphatase, receptor type, N [Source:MGI Symbol;Acc:MGI:102765]	8.1	8.1	7.7	7.8	-1.3
ENSMUSG00000066677	Pydc3	pyrin domain containing 3 [Source:MGI Symbol;Acc:MGI:2442822]	10.0	9.9	10.3	10.5	1.3
ENSMUSG00000030359	Pzp	pregnancy zone protein [Source:MGI Symbol;Acc:MGI:87854]	4.9	4.9	5.4	5.4	1.4
ENSMUSG00000003411	Rab3b	RAB3B, member RAS oncogene family [Source:MGI Symbol;Acc:MGI:1917158]	4.4	4.3	5.2	5.3	1.8
ENSMUSG00000029602	Rasal1	RAS protein activator like 1 (GAP1 like) [Source:MGI Symbol;Acc:MGI:1330842]	4.7	4.7	4.0	3.9	-1.7
ENSMUSG00000025576	Rbfox3	RNA binding protein, fox-1 homolog (C. elegans) 3 [Source:MGI Symbol;Acc:MGI:106368]	6.3	6.4	6.8	6.9	1.4
ENSMUSG00000029136	Rbks	ribokinase [Source:MGI Symbol;Acc:MGI:1918586]	7.0	6.8	7.4	7.6	1.5
ENSMUSG00000032454	Rbp2	retinol binding protein 2, cellular [Source:MGI Symbol;Acc:MGI:97877]	4.1	4.0	5.3	5.4	2.4
ENSMUSG00000048899	Rimkla	ribosomal modification protein rimK-like family member A [Source:MGI Symbol;Acc:MGI:3040686]	4.9	5.0	4.4	4.6	-1.4
ENSMUSG00000046215	Rprml	reprimo-like [Source:MGI Symbol;Acc:MGI:2144486]	6.2	6.2	7.0	7.3	1.9
ENSMUSG00000074115	Saa1	serum amyloid A 1 [Source:MGI Symbol;Acc:MGI:98221]	2.9	2.4	5.0	5.5	6.2
ENSMUSG00000031665	Sall1	sal-like 1 (Drosophila) [Source:MGI Symbol;Acc:MGI:1889585]	4.2	4.4	5.3	5.8	2.4
ENSMUSG00000070304	Scn2b	sodium channel, voltage-gated, type II, beta [Source:MGI Symbol;Acc:MGI:106921]	6.3	6.2	5.9	5.7	-1.4
ENSMUSG00000005696	Sh2d1a	SH2 domain containing 1A [Source:MGI Symbol;Acc:MGI:1328352]	3.6	3.8	5.4	4.9	2.8

ENSMUSG00000051504	Siglech	sialic acid binding Ig-like lectin H [Source:MGI Symbol;Acc:MGI:2443256]	6.9	6.8	7.3	7.5	1.4
ENSMUSG00000070280	Slc22a14	solute carrier family 22 (organic cation transporter), member 14 [Source:MGI Symbol;Acc:MGI:2685974]	4.7	4.6	5.1	5.2	1.4
ENSMUSG00000029175	Slc35f6	solute carrier family 35, member F6 [Source:MGI Symbol;Acc:MGI:1922169]	11.3	11.3	11.7	11.7	1.3
ENSMUSG00000006574	Slc4a1	solute carrier family 4 (anion exchanger), member 1 [Source:MGI Symbol;Acc:MGI:109393]	9.3	10.1	7.1	6.3	-7.8
ENSMUSG00000041644	Slc5a12	solute carrier family 5 (sodium/glucose cotransporter), member 12 [Source:MGI Symbol;Acc:MGI:2138890]	5.0	5.6	6.8	6.7	2.8
ENSMUSG00000025938	Slco5a1	solute carrier organic anion transporter family, member 5A1 [Source:MGI Symbol;Acc:MGI:2443431]	8.5	8.5	8.1	8.2	-1.3
ENSMUSG00000036867	Smad6	SMAD family member 6 [Source:MGI Symbol;Acc:MGI:1336883]	12.6	12.6	13.0	13.1	1.3
ENSMUSG00000025889	Snca	synuclein, alpha [Source:MGI Symbol;Acc:MGI:1277151]	8.7	9.3	7.6	7.4	-2.8
ENSMUSG00000026532	Spta1	spectrin alpha, erythrocytic 1 [Source:MGI Symbol;Acc:MGI:98385]	6.6	7.1	5.6	5.5	-2.5
ENSMUSG00000017400	Stac2	SH3 and cysteine rich domain 2 [Source:MGI Symbol;Acc:MGI:2144518]	7.1	7.2	6.2	6.3	-1.8
ENSMUSG00000031027	Stk33	serine/threonine kinase 33 [Source:MGI Symbol;Acc:MGI:2152419]	9.0	9.0	9.4	9.4	1.3
ENSMUSG00000041479	Syt15	synaptotagmin XV [Source:MGI Symbol;Acc:MGI:2442166]	8.4	8.2	8.9	9.1	1.7
ENSMUSG00000001983	Taco1	translational activator of mitochondrially encoded cytochrome c oxidase I [Source:MGI Symbol;Acc:MGI:1917457]	9.1	8.9	8.6	8.6	-1.3
ENSMUSG00000002781	Tmem143	transmembrane protein 143 [Source:MGI Symbol;Acc:MGI:1917459]	8.8	8.7	9.1	9.1	1.3
ENSMUSG00000049526	Tmem202	transmembrane protein 202 [Source:MGI Symbol;Acc:MGI:1921143]	4.8	4.7	5.3	5.3	1.5
ENSMUSG00000048572	Tmem252	transmembrane protein 252 [Source:MGI Symbol;Acc:MGI:3583948]	11.7	11.7	11.0	10.9	-1.8
ENSMUSG00000005628	Tmod4	tropomodulin 4 [Source:MGI Symbol;Acc:MGI:1355285]	6.0	5.9	5.4	5.5	-1.4

ENSMUSG00000032268	Tmprss5	transmembrane protease, serine 5 (spinesin) [Source:MGI Symbol;Acc:MGI:1933407]	5.6	5.6	5.2	5.1	-1.4
ENSMUSG00000073400	Trim10	tripartite motif-containing 10 [Source:MGI Symbol;Acc:MGI:1338757]	6.6	7.4	4.6	4.3	-6.0
ENSMUSG00000035601	Trmt10b	tRNA methyltransferase 10B [Source:MGI Symbol;Acc:MGI:1917184]	8.5	8.5	8.8	8.9	1.3
ENSMUSG00000025272	Tro	trophinin [Source:MGI Symbol;Acc:MGI:1928994]	7.4	7.5	7.8	7.8	1.3
ENSMUSG00000022510	Trp63	transformation related protein 63 [Source:MGI Symbol;Acc:MGI:1330810]	5.8	6.0	5.3	5.3	-1.5
ENSMUSG00000027716	Trpc3	transient receptor potential cation channel, subfamily C, member 3 [Source:MGI Symbol;Acc:MGI:109526]	8.6	8.7	8.1	7.9	-1.6
ENSMUSG00000022388	Ttll8	tubulin tyrosine ligase-like family, member 8 [Source:MGI Symbol;Acc:MGI:1922902]	6.5	6.5	6.2	6.1	-1.3
ENSMUSG00000062380	Tubb3	tubulin, beta 3 class III [Source:MGI Symbol;Acc:MGI:107813]	7.7	7.7	7.1	7.3	-1.4
ENSMUSG00000034485	Uaca	uveal autoantigen with coiled-coil domains and ankyrin repeats [Source:MGI Symbol;Acc:MGI:1919815]	13.0	12.9	12.6	12.5	-1.3
ENSMUSG00000027300	Ubox5	U box domain containing 5 [Source:MGI Symbol;Acc:MGI:2154658]	8.6	8.7	9.1	9.2	1.4
ENSMUSG00000020099	Unc5b	unc-5 homolog B (C. elegans) [Source:MGI Symbol;Acc:MGI:894703]	11.8	11.7	11.4	11.3	-1.3
ENSMUSG00000037989	Wnk2	WNK lysine deficient protein kinase 2 [Source:MGI Symbol;Acc:MGI:1922857]	7.5	7.4	6.9	6.6	-1.6
ENSMUSG00000048728	Zfp454	zinc finger protein 454 [Source:MGI Symbol;Acc:MGI:2679253]	6.2	6.1	6.7	6.7	1.4
ENSMUSG00000055202	Zfp811	zinc finger protein 811 [Source:MGI Symbol;Acc:MGI:2682944]	4.4	4.3	5.5	5.1	2.0

Table S3. List of Fluidigm and Biolegend antibodies used for mass immunophenotyping

	Metal	Target
1	89Y	CD45
2	141Pr	CCR6
3	142Nd	CD19
4	143Nd	anti-CX3CR1-biotin + anti-biotin-143
5	144Nd	CD11b
6	145Nd	CD4
7	146Nd	IgD
8	147Sm	CD11c
9	148Nd	CD16
10	149Sm	CD127
11	150Nd	CD86
12	151Eu	HLA-DR
13	152Sm	CD21
14	153Eu	CXCR5
15	154Sm	TIGIT
16	155Gd	PD-1
17	156Gd	CXCR3
18	158Gd	CCR4
19	159Tb	CCR7
20	160Gd	CD14
21	162Dy	CD27
22	163Dy	CD34
23	164Dy	CD45RO
24	165Ho	anti-CD133-PE + anti-PE
25	166Er	CD24
26	167Er	CD38
27	168Er	CD8
28	169Tm	CD25
29	170Er	CD3
30	171Yb	CD68
31	172Yb	IgM
32	173Yb	CXCR4
33	174Yb	anti-CCR2-FITC + anti-FITC
34	175Lu	Rabbit anti-BTK + anti-rabbit IgG
35	176Yb	anti-CD28-APC + anti-APC
36	191/193	Nucleic acid Intercalator
37	198Pt	Cisplatin

Table S4. Immuno cell type surface markers used in the CyTOF study

POPULATION	PHENOTYPE
CD16+ NK Cells	CD16+ CD14- HLADR-
CD16- NK Cells	CD16- CD14- HLADR-
Non-Classical Monocytes	CD16+CD14^{lo}HLA-DR+CD11b+CD4^{lo}
Classical Monocytes	CD16-CD14+HLA-DR+CD11b^{hi}CD4^{lo}
Island 1	CD25+CD38+CD45RO+
mDCs	CD14-CD16-HLA-DR+CD11c+CD4^{lo}
pDCs	CD14-CD16-HLA-DR+CD11c-CD4^{lo}
Plasmablasts	CD19+CD38^{HI}CD27^{HI}
Island 5	CD38+CD27+TIGIT+CD16^{lo}
Island 6	CD38+CD27+CXCR3+CD11c+CD11b+
Island 7	CD25+CD27+CCR4+CD127+
Naive B Cells	CD19+CD27-IgD+
US Memory B Cells	CD19+CD27-IgD+CD133+CCR7+
CD133+ Island 2	CD19+CD21+IgD+IgM+CD133+
CD133+ Island 3	CD19+CD21+IgD+IgM+CCR6+CD133+
T and B Cell Doublets	CD3+CD8+CD19+
DP T Cells (Memory)	CD3+CD4+CD8+CD45RO+CCR7-
DP T Cells (2) (Naive)	CD3+CD4+CD8+CD45RO-CCR7+
HSCs	CD133+CD34+CD38+CD45^{lo}
CD8+ Tnaive Cels	CD3+CD8+CD45RO-CCR7+
CD8+ Tcm Cells	CD3+CD8+CD45RO+CCR7+
CS Memory B Cells	CD19+CD27+IgD-
CD8+ Tem Cells	CD3+CD8+CD45RO+CCR7-
CD8+ Temra Cells	CD3+CD8+CD45RO-CCR7-
CD4+ Tnaive Cells	CD3+CD4+CD45RO-CCR7+
CD4+ Tcm Cells	CD3+CD4+CD45RO+CCR7+
CD4+ Tem Cells	CD3+CD4+CD45RO+CCR7-
Tfh Cells	CD3+CD4+CXCR5+PD1+
Naive DN T Cells (TCRgd)	CD3+CD4-CD8-CD45RO-CCR7+
Memory DN T Cells (TCRgd)	CD3+CD4-CD8-CD45RO+CCR7-
CD8+ Tem Cells (2)	CD3+CD8+CD45RO+CCR7-
CD8+ Temra Cells (2)	CD3+CD8+CD45RO-CCR7-

Table S5. Demographics of volunteers/patients served in the CyTOF study

Clinical subjects	Age	Sex
Healthy Volunteer 1	7	Male
Healthy Volunteer 2	13	Male
PAH patient with KCNK3 mutation	9	Male
PAH patient with KCNK3 mutation	11	Male

Table S6. List of antibodies used in conventional flow cytometry

ThermoFisher Scientific	Catalog details
eBioscience™ Mouse Hematopoietic Lineage Biotin Panel	88-7774-75
eBioscience™ Streptavidin eFluor™ 450 Conjugate	48-4317-82
Ly-6A/E (Sca-1) Monoclonal Antibody (D7), PE, eBioscience™	12-5981-82
CD117 (c-Kit) Monoclonal Antibody (2B8), APC-eFluor 780, eBioscience™	47-1171-82
CD135 (Flt3) Monoclonal Antibody (A2F10), PE-Cyanine5, eBioscience™	15-1351-82
CD34 Monoclonal Antibody (RAM34), eFluor 660, eBioscience™	50-0341-82
CD16/CD32 Monoclonal Antibody (93), PE-Cyanine7, eBioscience™	25-0161-82
Ly-6G/Ly-6C Monoclonal Antibody (RB6-8C5), PE-Cyanine7, eBioscience™	25-5931-82
CD11b Monoclonal Antibody (M1/70), PE, eBioscience™	12-0112-82
CD4 Monoclonal Antibody (RM4-5), eFluor 450, eBioscience™	48-0042-82
CD8a Monoclonal Antibody (53-6.7), APC-eFluor 780, eBioscience™	47-0081-82
CD45R (B220) Monoclonal Antibody (RA3-6B2), APC, eBioscience™	17-0452-82

Table S7. Surface Master Mix

Conjugate	Target	Clone	Vendor	Volume/test (uL)
89Y	CD45	HI30	Fluidigm	0.25
141Pr	CCR6	G034E3	Fluidigm	1.25
142Nd	CD19	HIB19	Fluidigm	0.25
Biotin	CX3CR1	2A91	Biolegend	2
144Nd	CD11b	ICRF44	Fluidigm	0.1
145Nd	CD4	RPA-T4	Fluidigm	0.25
146Nd	IgD	IA6-2	Fluidigm	0.1
147Sm	CD11c	Bu15	Fluidigm	0.25
148Nd	CD16	3G8	Fluidigm	0.25
149Sm	CD127	A019D5	Fluidigm	2.5
150Nd	CD86	IT2.2	Fluidigm	1.5
151Eu	HLA-DR	G46-6	Fluidigm	0.1
152Sm	CD21	BL13	Fluidigm	1
153Eu	CXCR5	RF8B2	Fluidigm	1
154Sm	TIGIT	MBSA43	Fluidigm	1
155Gd	PD-1	EH12.2H7	Fluidigm	2
156Gd	CXCR3	G025H7	Fluidigm	2
158Gd	CCR4	L291H4	Fluidigm	1
159Tb	CCR7	G043H7	Fluidigm	0.75
160Gd	CD14	M5E2	Fluidigm	1
162Dy	CD27	L128	Fluidigm	0.5
163Dy	CD34	581	Fluidigm	1
164Dy	CD45RO	UCHL1	Fluidigm	0.5
PE	CD133	clone 7	Biolegend	2
166Er	CD24	ML5	Fluidigm	1.5
167Er	CD38	HIT2	Fluidigm	0.5
168Er	CD8	SKI1	Fluidigm	0.05
169Tm	CD25	2A3	Fluidigm	0.5
170Er	CD3	UCHT1	Fluidigm	0.25
172Yb	IgM	MHM-88	Fluidigm	0.1
173Yb	CXCR4	12G5	Fluidigm	1
APC	CD28	CD28.2	Biolegend	2
PBS/1% BSA				71.55
**Total Per Test**				**100**

Table S8. Mouse Cytokine Array coordinates

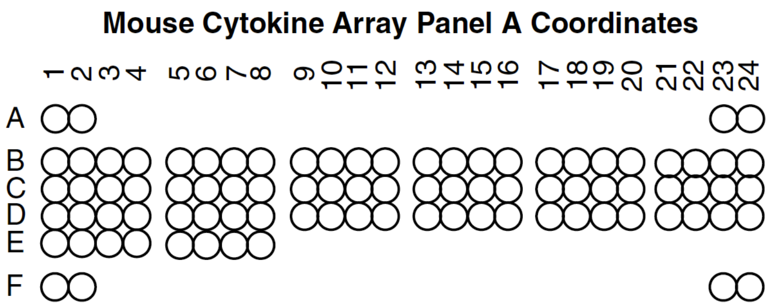

Coordinate	Target	Alternate Nomenclature
A1, A2	Reference Spot	-
A23, A24	Reference Spot	-
B1, B2	BLC	CXCL13/BCA-1
B3, B4	C5/C5a	Complement Component 5a
B5, B6	G-CSF	-
B7, B8	GM-CSF	-
B9, B10	I-309	CCL1/TCA-3
B11, B12	Eotaxin	CCL11
B13, B14	sICAM-1	CD54
B15, B16	IFN-γ	-
B17, B18	IL-1α	IL-1F1
B19, B20	IL-1β	IL-1F2
B21, B22	IL-1ra	IL-1F3
B23, B24	IL-2	-
C1, C2	IL-3	-
C3, C4	IL-4	-
C5, C6	IL-5	-
C7, C8	IL-6	-
C9, C10	IL-7	-
C11, C12	IL-10	-
C13, C14	IL-13	-
C15, C16	IL-12 p70	-
C17, C18	IL-16	-
C19, C20	IL-17	-
C21, C22	IL-23	-
C23, C24	IL-27	-

D1, D2	IP-10	CXCL10/CRG-2
D3, D4	I-TAC	CXCL11
D5, D6	KC	CXCL1
D7, D8	M-CSF	-
D9, D10	JE	CCL2/MCP-1
D11, D12	MCP-5	CCL12
D13, D14	MIG	CXCL9
D15, D16	MIP-1α	CCL3
D17, D18	MIP-1β	CCL4
D19, D20	MIP-2	CXCL2
D21, D22	RANTES	CCL5
D23, D24	SDF-1	CXCL12
E1, E2	TARC	CCL17
E3, E4	TIMP-1	-
E5, E6	TNF-α	-
E7, E8	TREM-1	-
F1, F2	Reference Spot	-
F23, F24	PBS (Negative Control)	Control (-)

Table S9. Secondary Surface Master Mix

Conjugate	Target	Clone	Vendor	Volume/test (uL)
143Nd	Biotin	ID4-C5	Fluidigm	2
165Ho	PE	PE001	Fluidigm	1
176Yb	APC	APC003	Fluidigm	1
PBS/1% BSA				96
**Total Per Test**				**100**

Table S10. Primary Intracellular Master Mix

Conjugate	Target	Clone	Vendor	Volume/test (uL)
171Yb	CD68	Y1/82A	Fluidigm	1
FITC	CCR2	K036C2	Biolegend	1
---	Rabbit anti-BTK	D3H5	Cell Signaling	0.5
Fix/Perm Buffer				97.5
**Total Per Test**				**100**

Table S11. Secondary Intracellular Master Mix

Conjugate	Target	Clone	Vendor	Volume/test (uL)
174Yb	anti-FITC	FIT-22	Fluidigm	0.2
175Lu	Goat anti-Rabbit IgG	Polyclonal	Fluidigm	0.5
Fix/Perm Buffer				99.3
**Total Per Test**				**100**
